# Supplementary figures and images for: NF1 regulates mesenchymal glioblastoma plasticity and aggressiveness through the AP-1 transcription factor FOSL1
Source: eLife. 2021 Aug 17;10:e64846. doi: 10.7554/eLife.64846 (PMC8370767; doi:10.7554/eLife.64846)

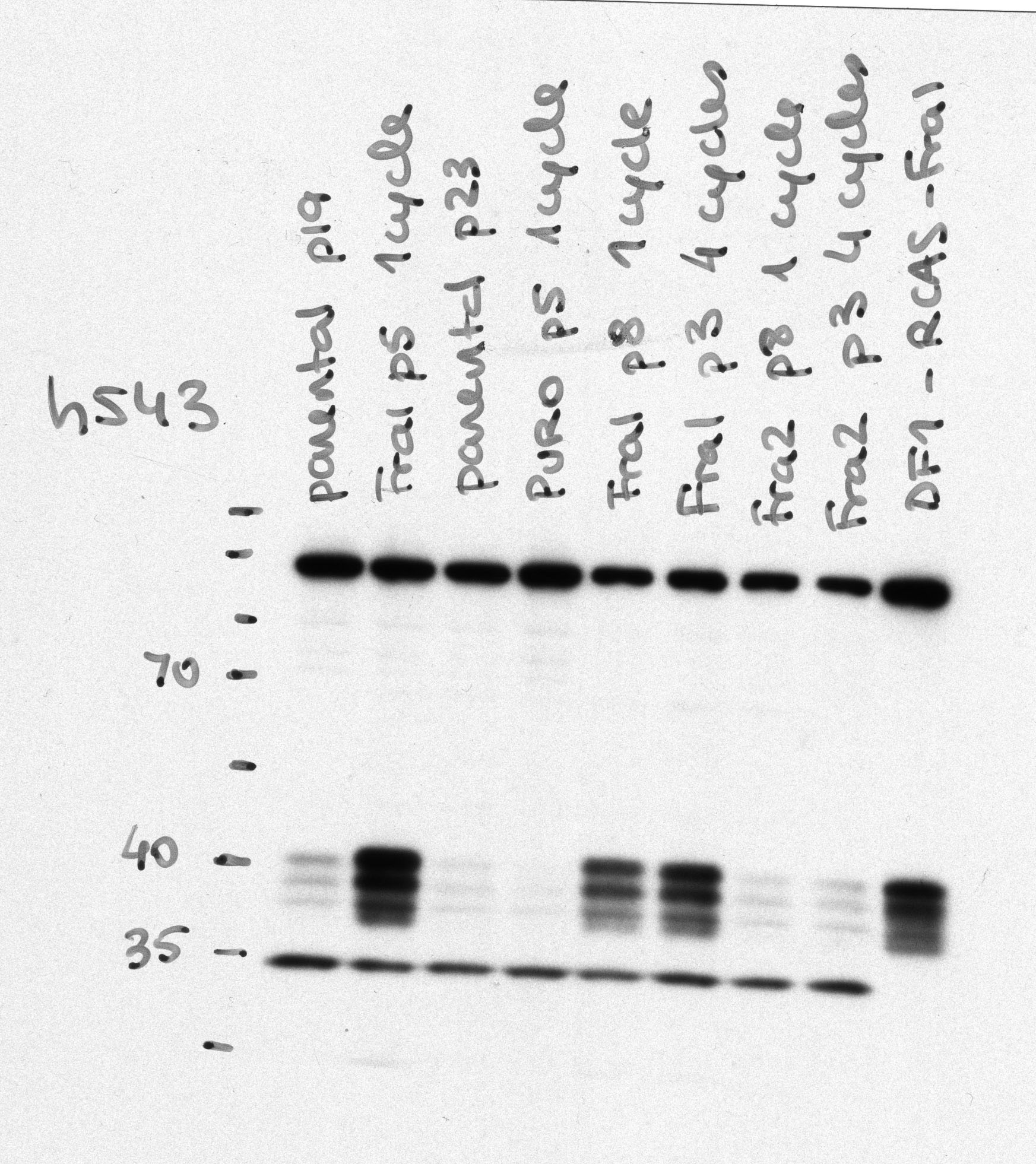

Supplement: Source data 1. [file elife-64846-data1.zip › Raw_images/Figure 7-figure supplement 1/Panel_H_h543_FOSL1_vin.jpg]

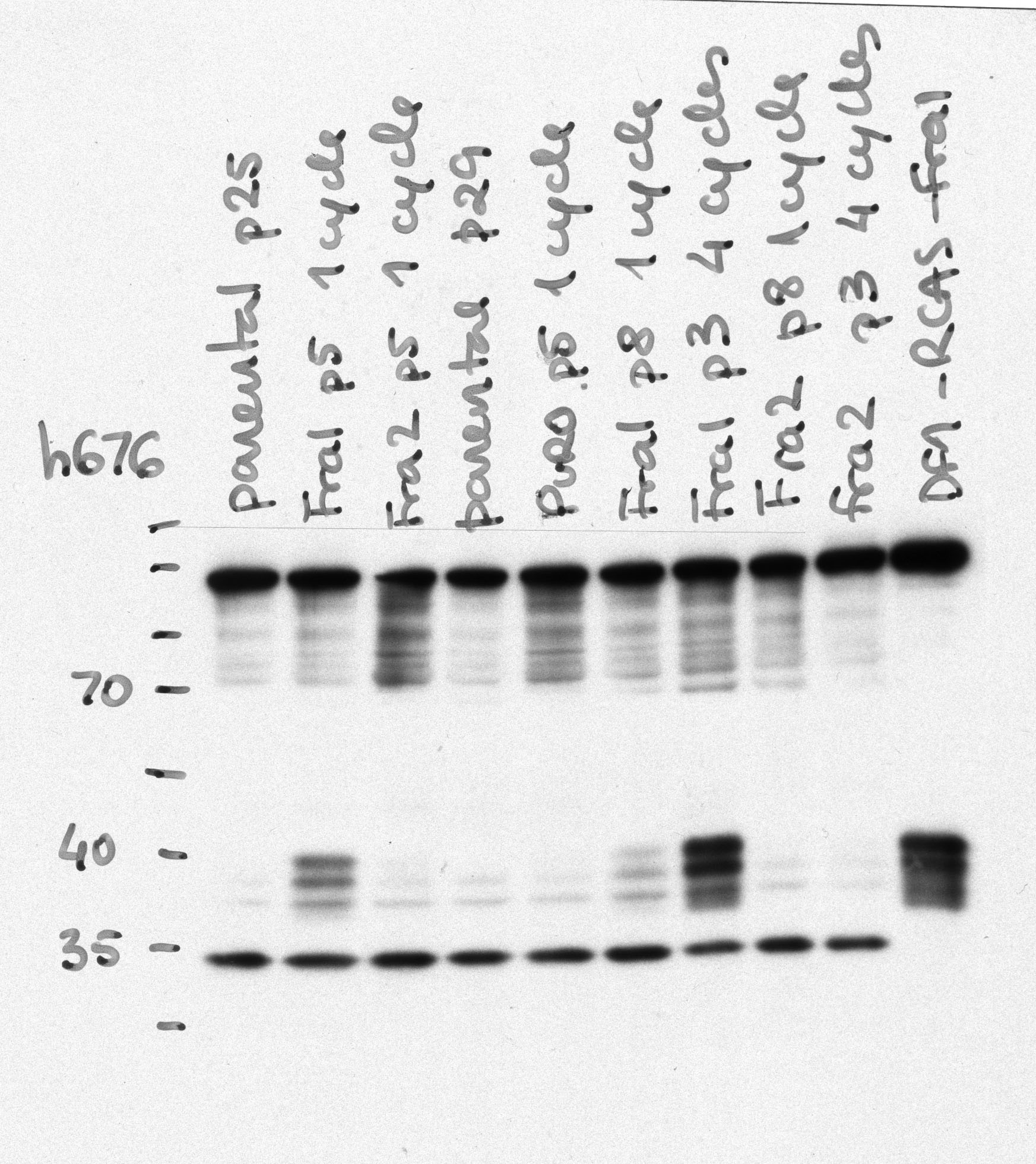

Supplement: Source data 1. [file elife-64846-data1.zip › Raw_images/Figure 7-figure supplement 1/Panel_H_h676_FOSL1_vin.jpg]

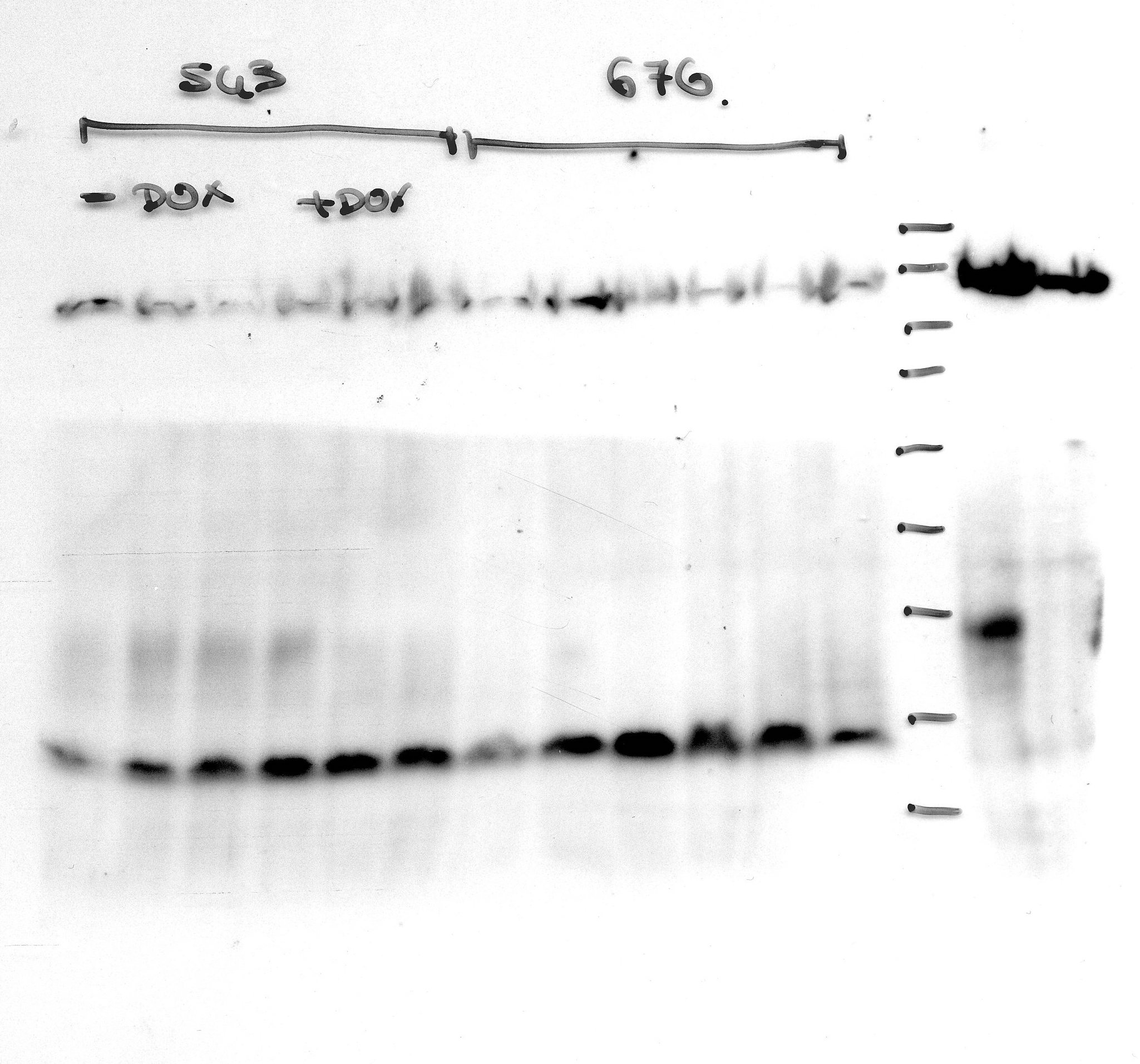

Supplement: Source data 1. [file elife-64846-data1.zip › Raw_images/Figure 7-figure supplement 1/Panel_J.jpg]

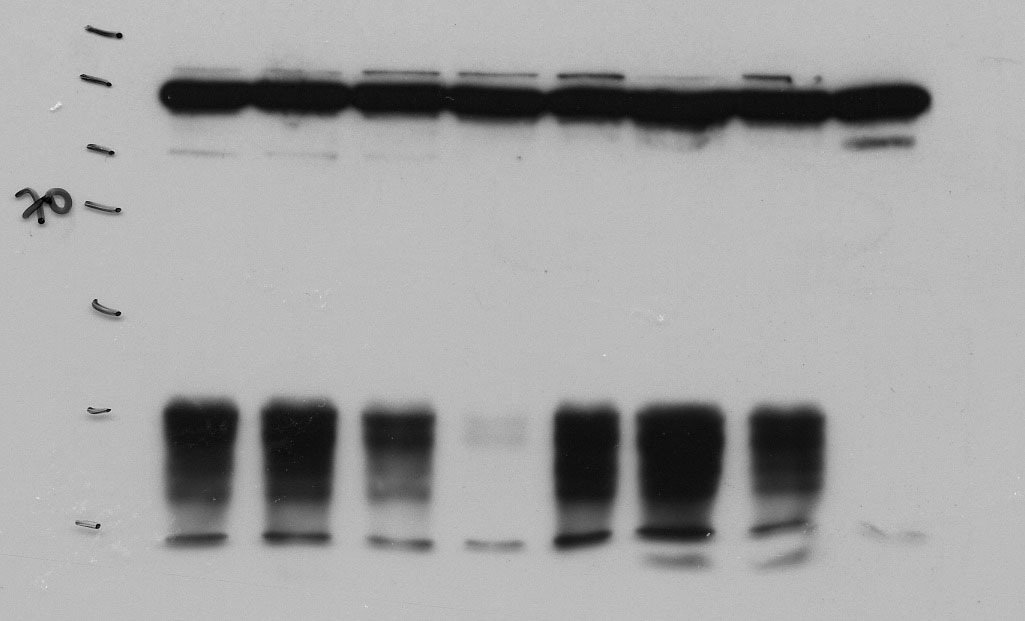

Supplement: Source data 1. [file elife-64846-data1.zip › Raw_images/Figure 7-figure supplement 1/Panel_A_FOSL1.jpg]

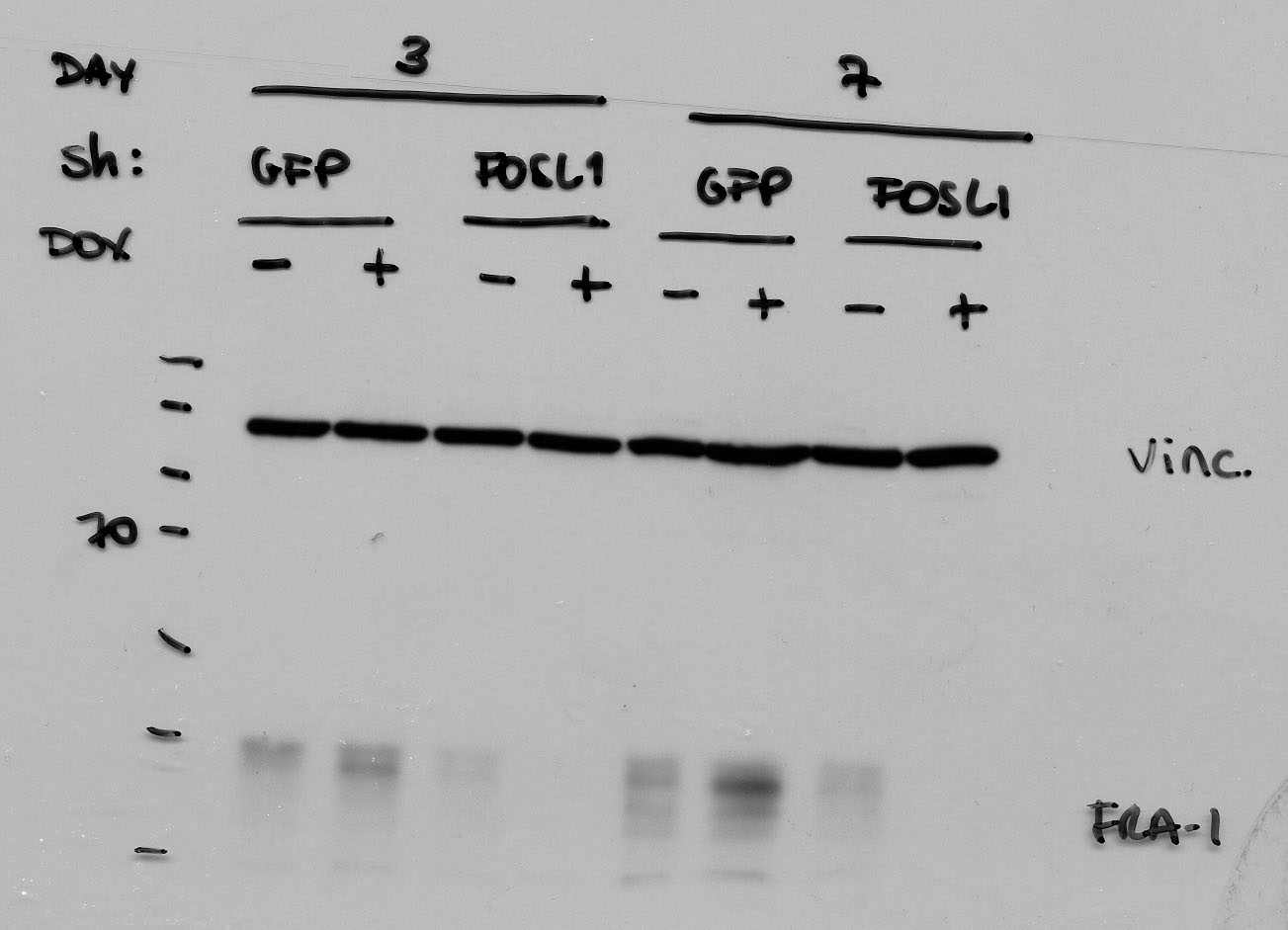

Supplement: Source data 1. [file elife-64846-data1.zip › Raw_images/Figure 7-figure supplement 1/Panel_A_vinculin.jpg]

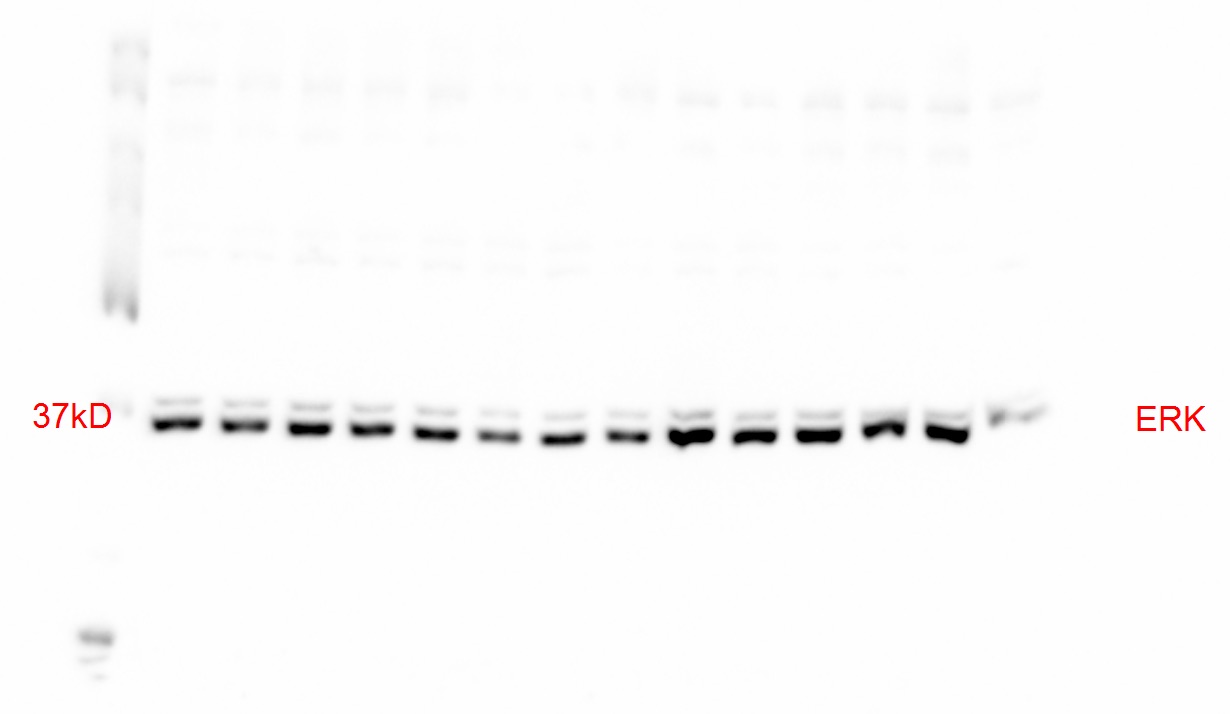

Supplement: Source data 1. [file elife-64846-data1.zip › Raw_images/Figure 2-figure supplement 1/Panel_A_ERK.jpg]

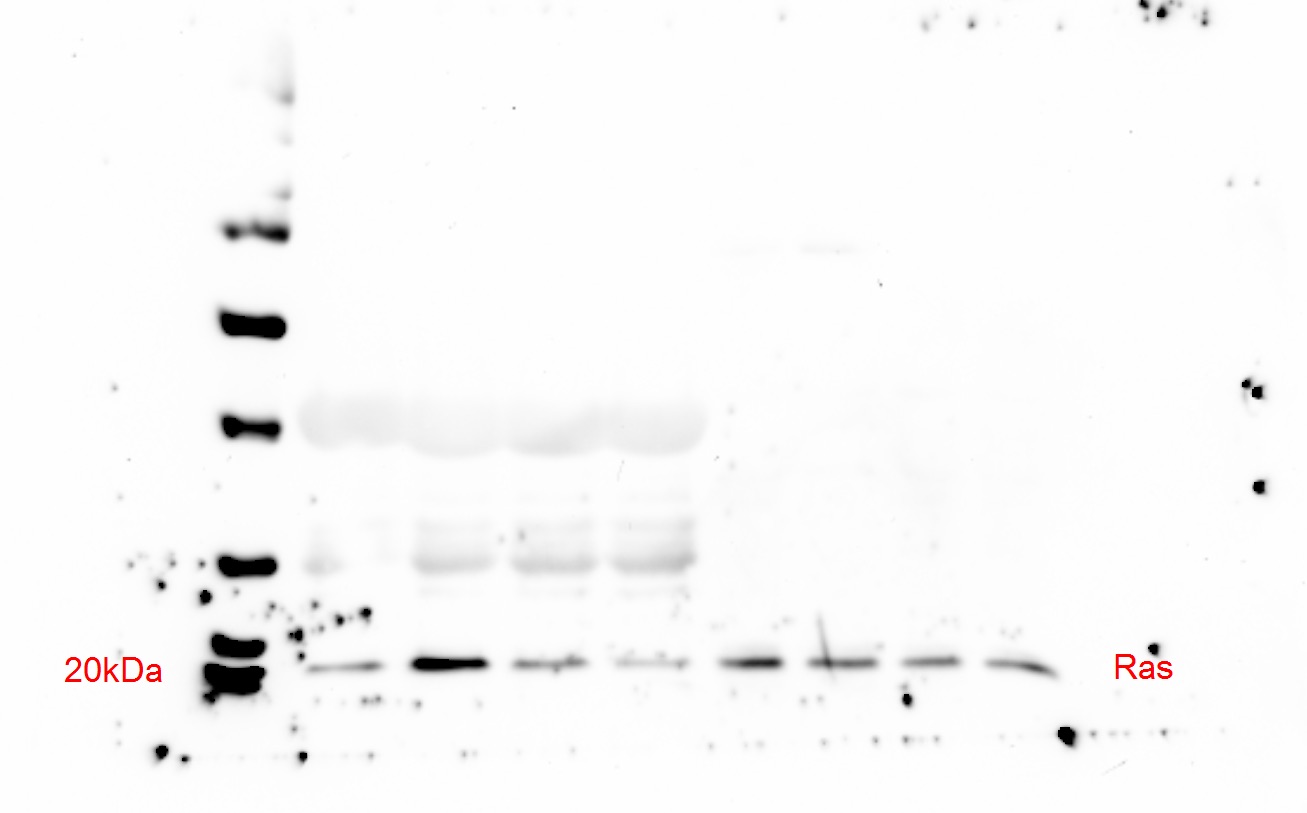

Supplement: Source data 1. [file elife-64846-data1.zip › Raw_images/Figure 2-figure supplement 1/Panel_C.jpg]

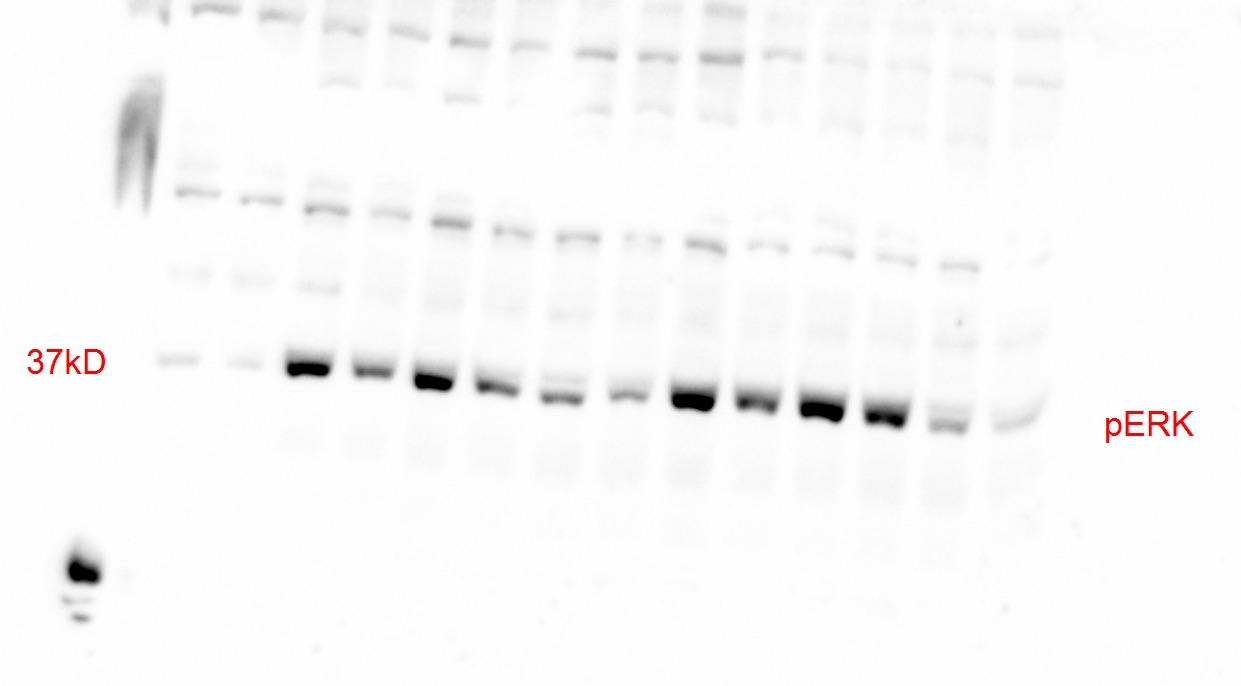

Supplement: Source data 1. [file elife-64846-data1.zip › Raw_images/Figure 2-figure supplement 1/Panel_A_pERK.jpg]

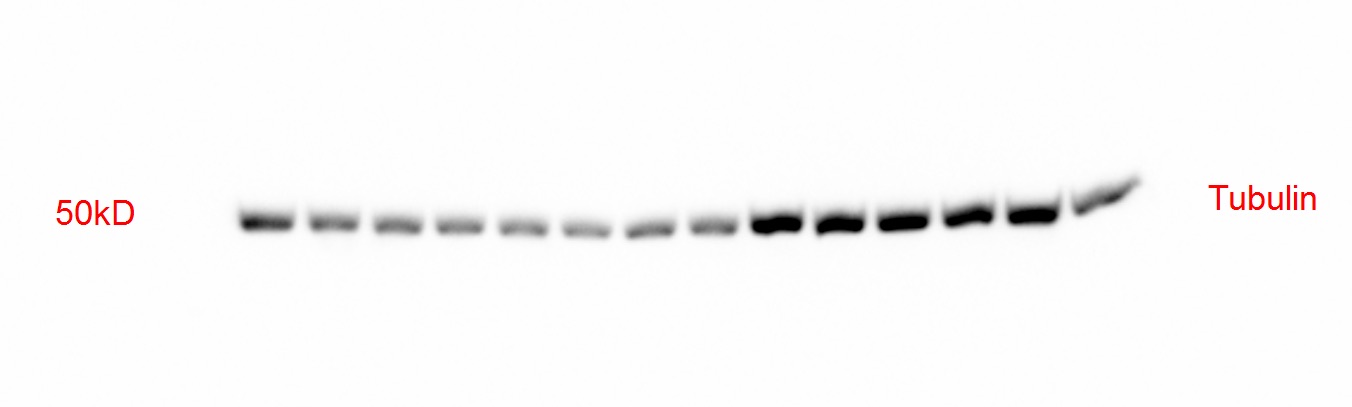

Supplement: Source data 1. [file elife-64846-data1.zip › Raw_images/Figure 2-figure supplement 1/Panel_A_Tubulin.jpg]

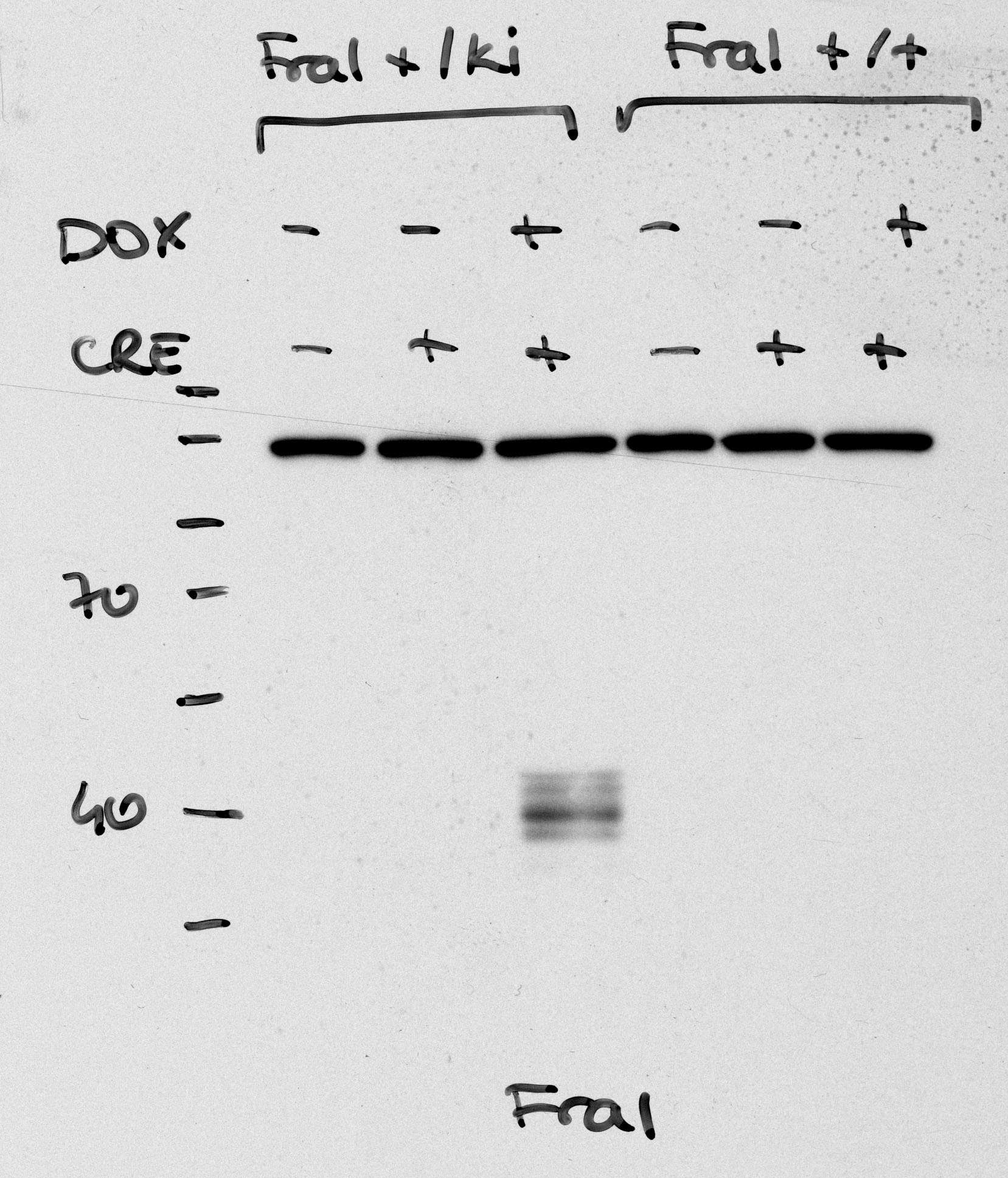

Supplement: Source data 1. [file elife-64846-data1.zip › Raw_images/Figure_6/Panel_A_Fra1_vinculin.jpg]

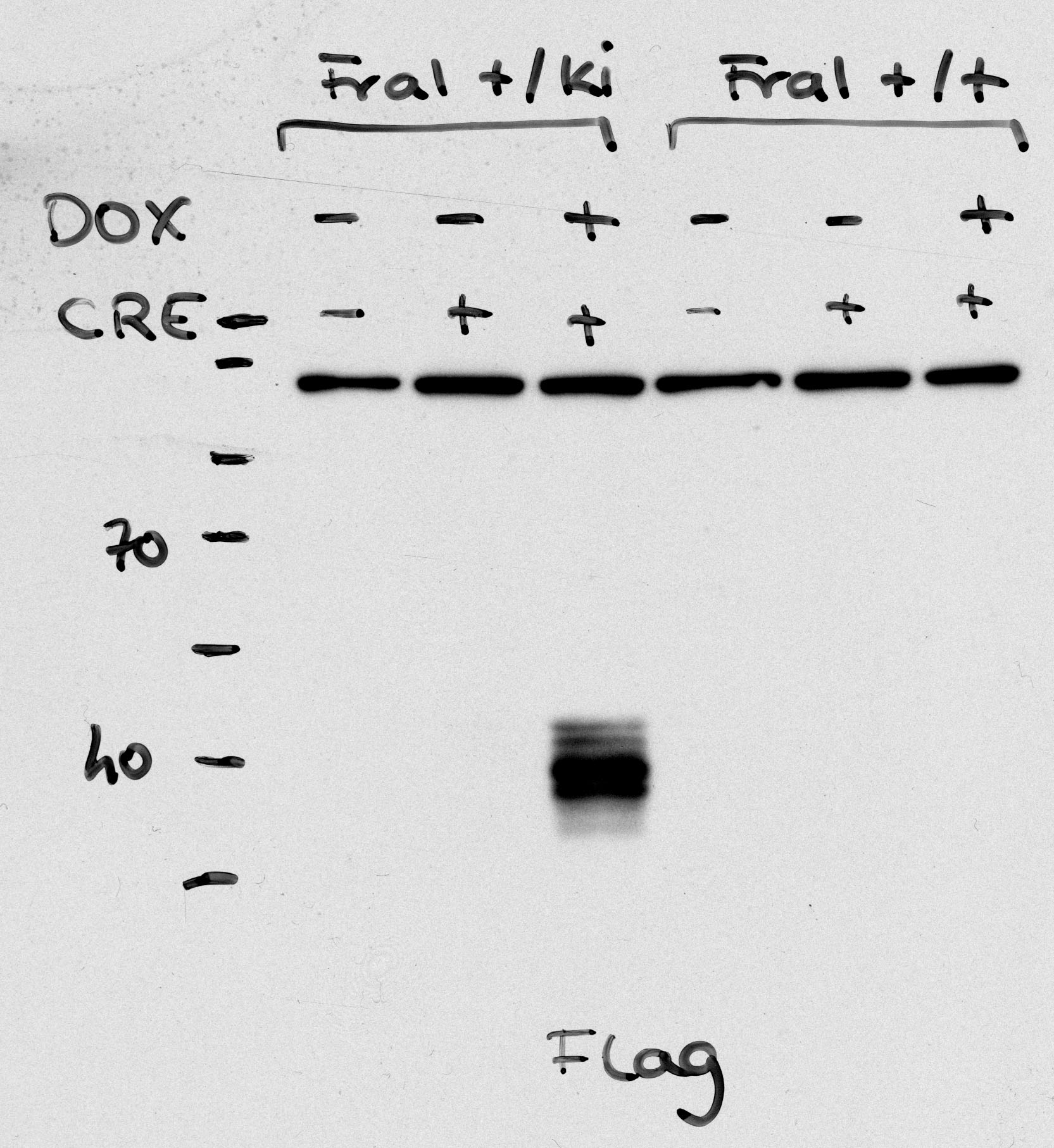

Supplement: Source data 1. [file elife-64846-data1.zip › Raw_images/Figure_6/Panel_A_Flag.jpg]

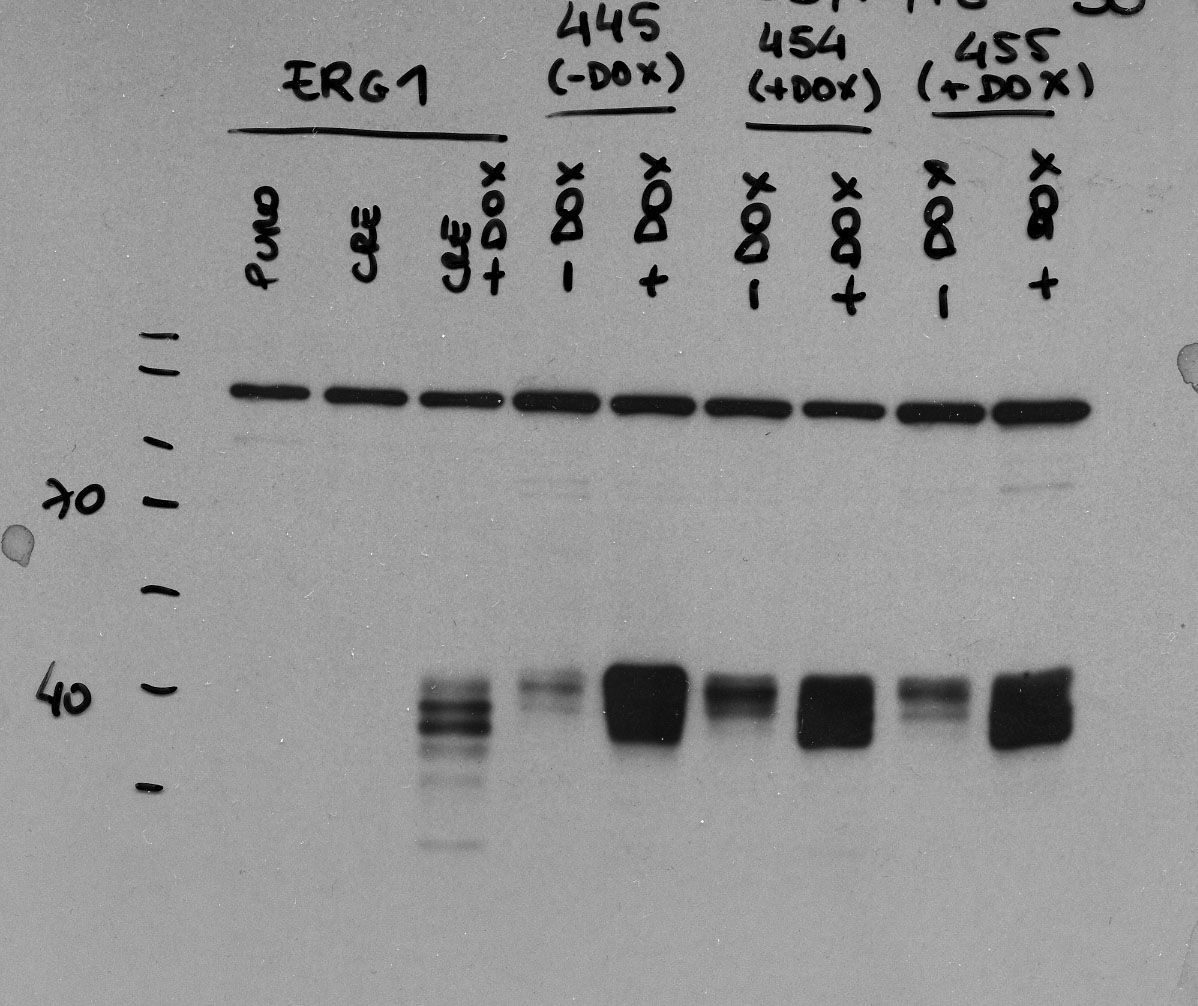

Supplement: Source data 1. [file elife-64846-data1.zip › Raw_images/Figure_6/Panel_E_H.jpg]

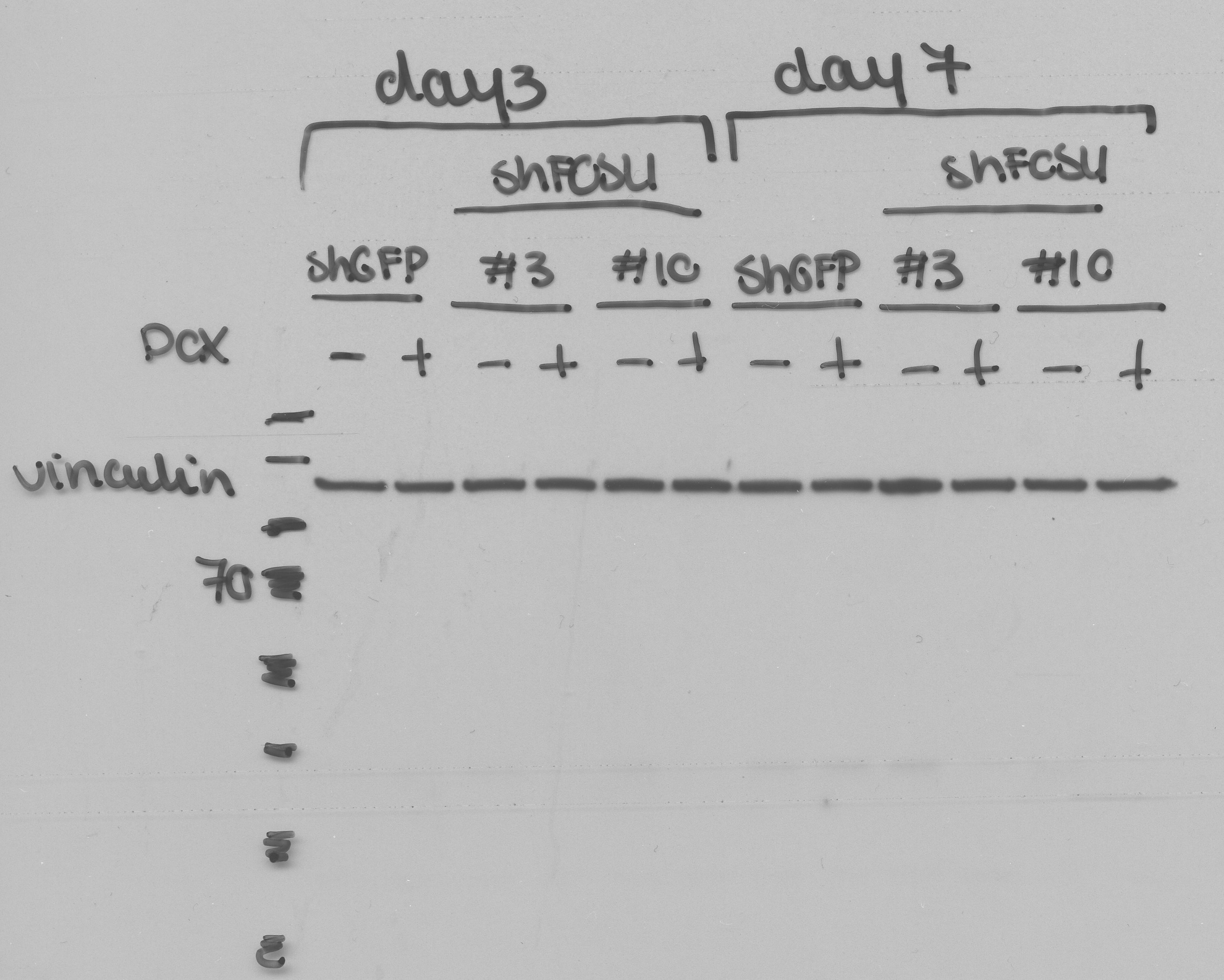

Supplement: Source data 1. [file elife-64846-data1.zip › Raw_images/Figure_7/Panel_B_vinculin.jpg]

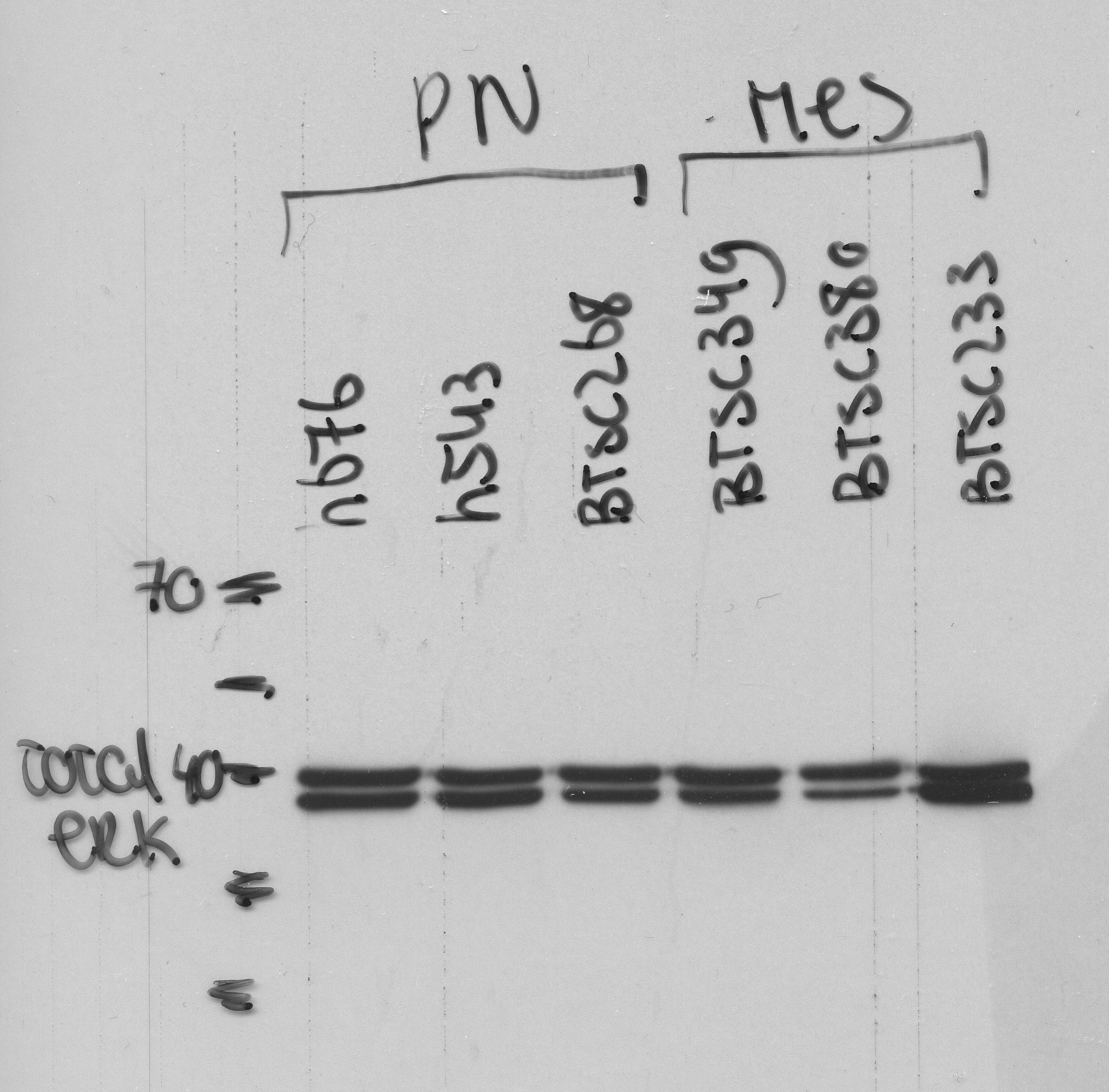

Supplement: Source data 1. [file elife-64846-data1.zip › Raw_images/Figure_7/Panel_A_Tot_ERK.jpg]

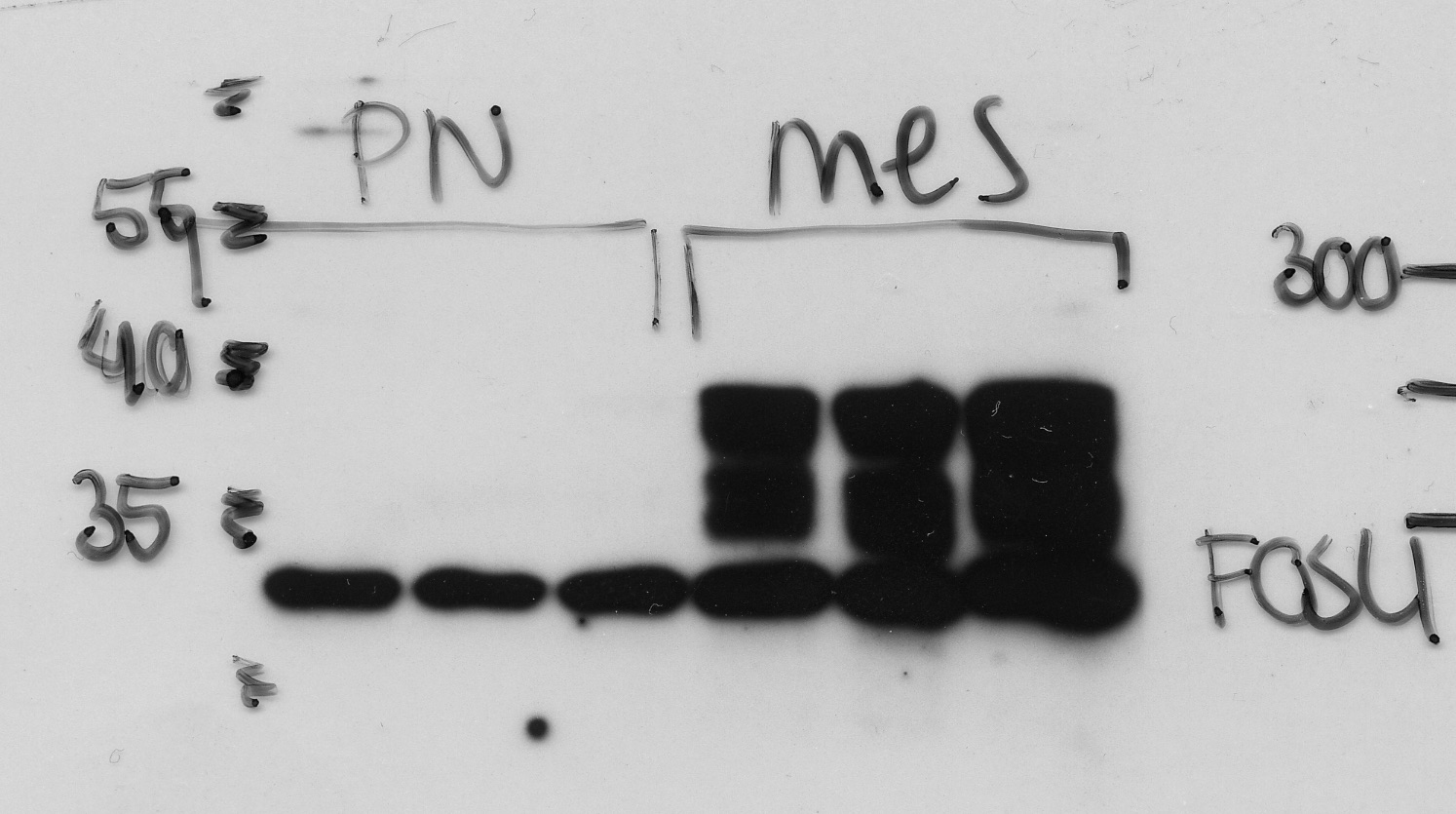

Supplement: Source data 1. [file elife-64846-data1.zip › Raw_images/Figure_7/Panel_A_FOSL1.jpg]

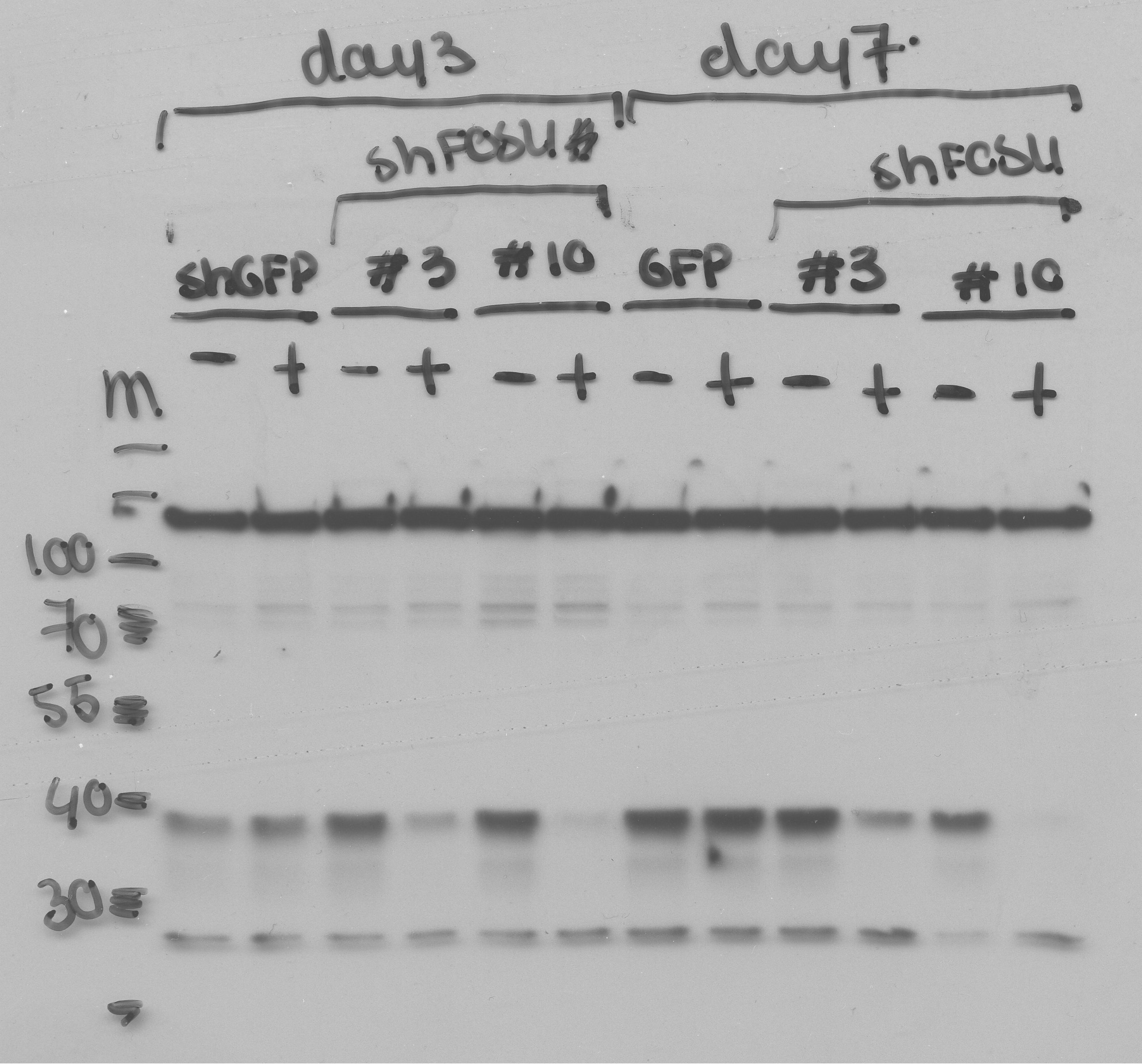

Supplement: Source data 1. [file elife-64846-data1.zip › Raw_images/Figure_7/Panel_B_FOSL1.jpg]

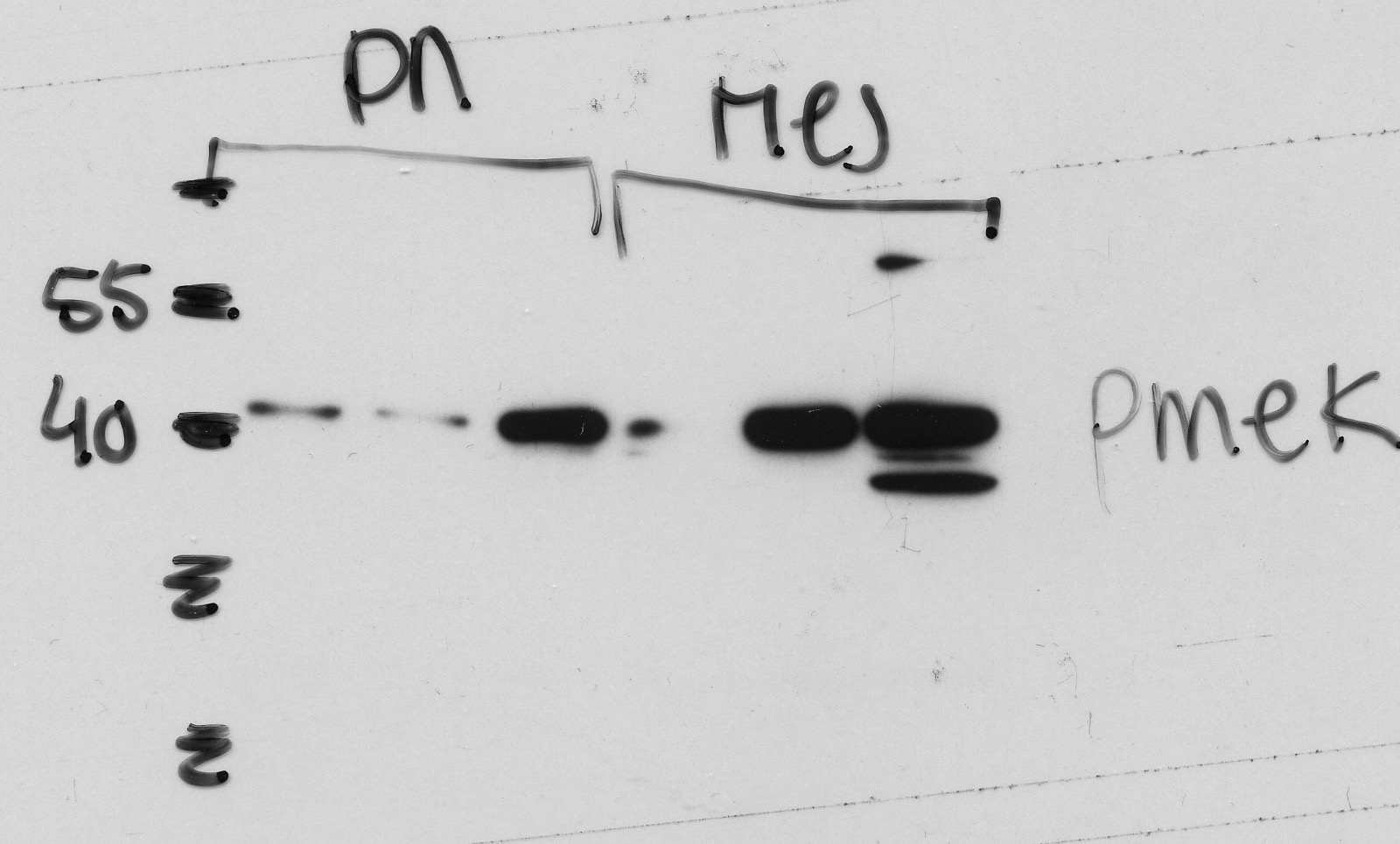

Supplement: Source data 1. [file elife-64846-data1.zip › Raw_images/Figure_7/Panel_A_pMEK.jpg]

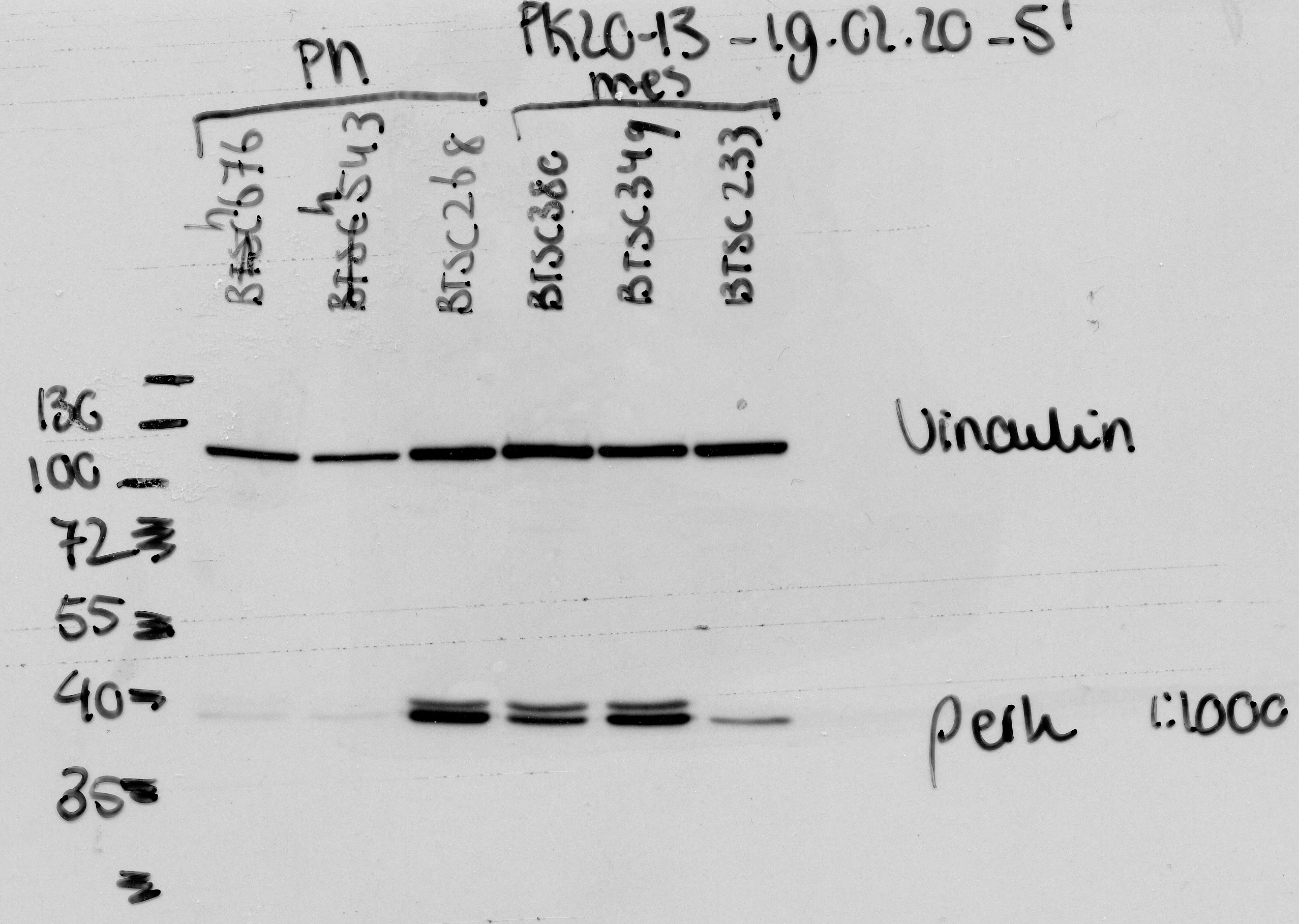

Supplement: Source data 1. [file elife-64846-data1.zip › Raw_images/Figure_7/Panel_A_pERK_vinculin.jpg]

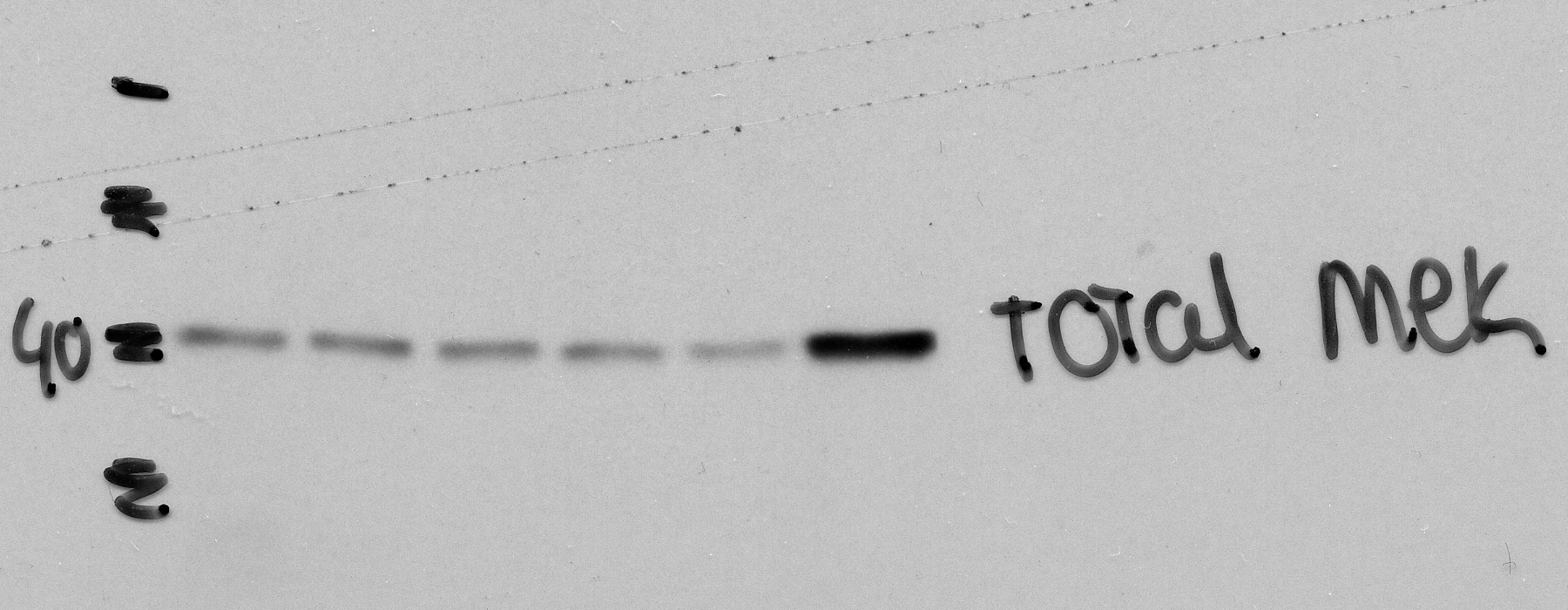

Supplement: Source data 1. [file elife-64846-data1.zip › Raw_images/Figure_7/Panel_A_TotMEK.jpg]

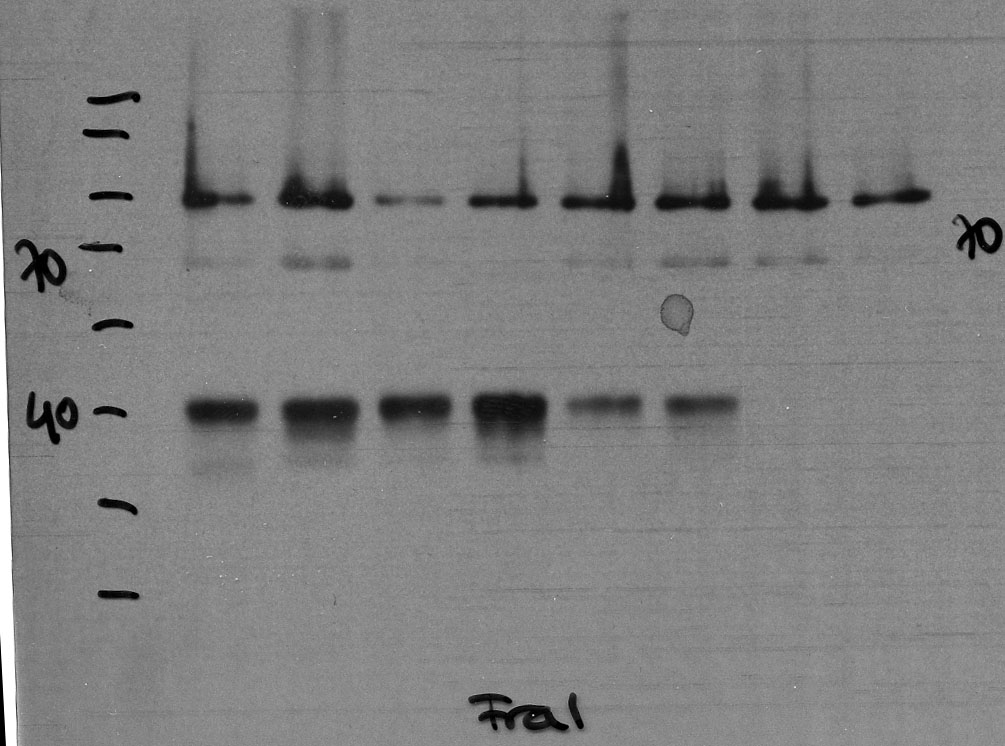

Supplement: Source data 1. [file elife-64846-data1.zip › Raw_images/Figure_5/Panel_G_Fra1]

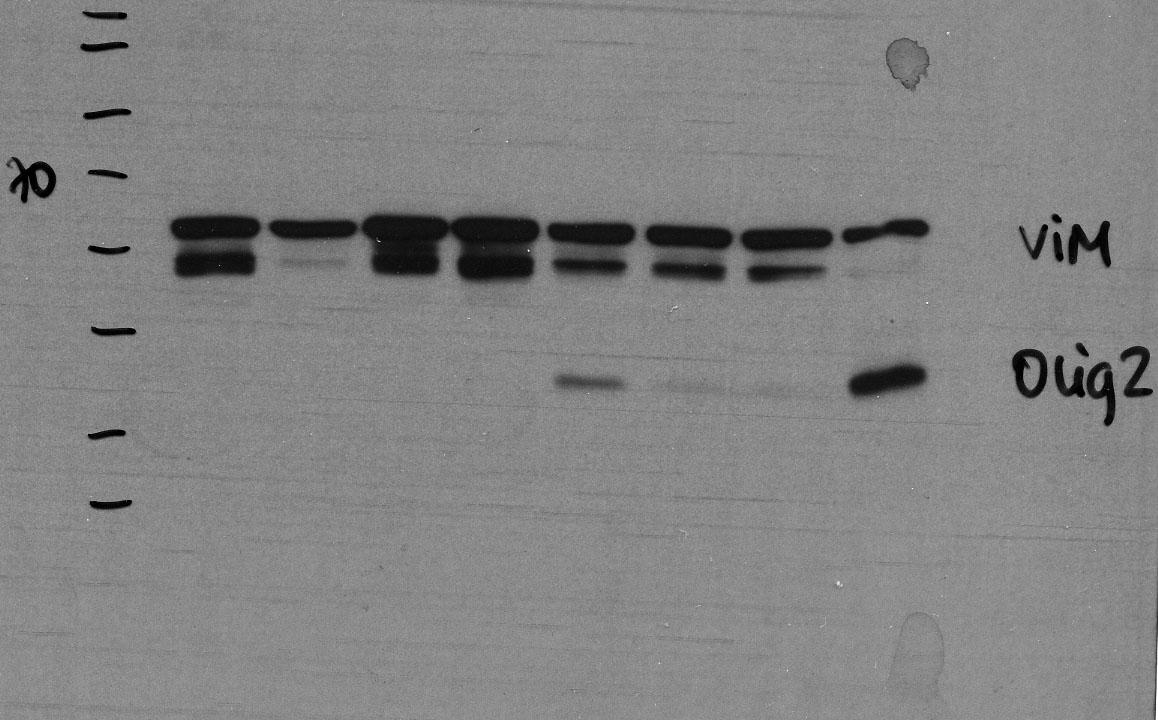

Supplement: Source data 1. [file elife-64846-data1.zip › Raw_images/Figure_5/Panel_G_Olig2_VIM]

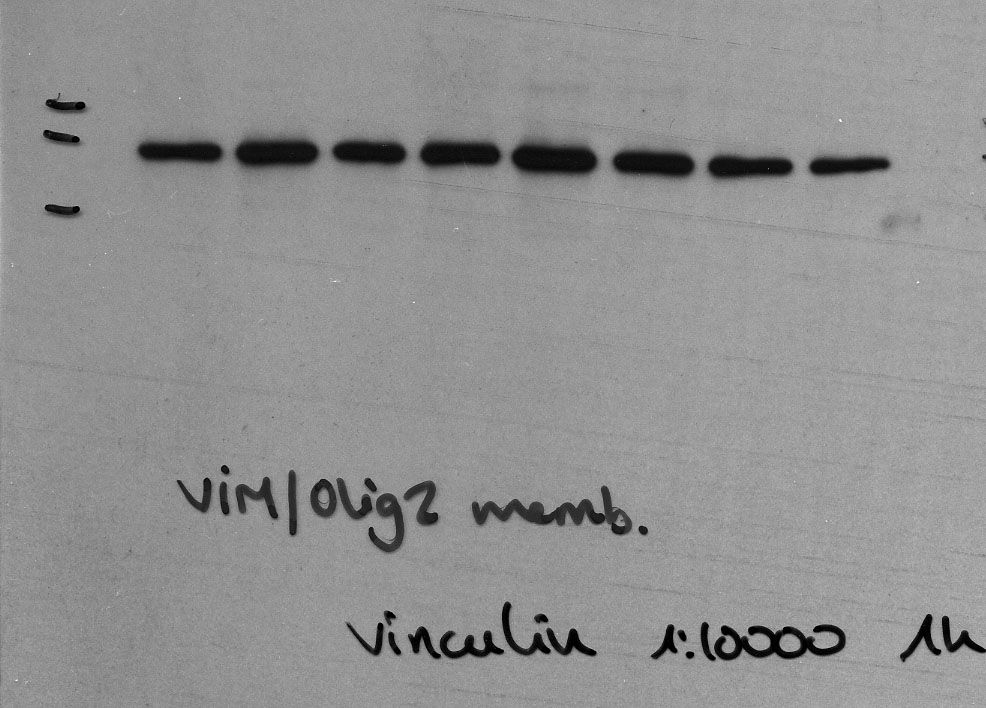

Supplement: Source data 1. [file elife-64846-data1.zip › Raw_images/Figure_5/Panel_G_vinculin]

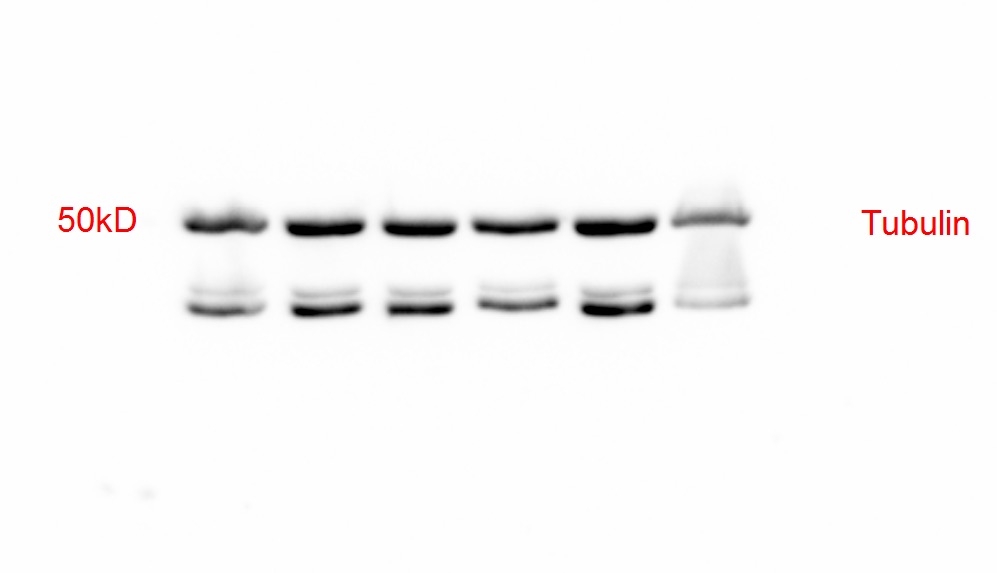

Supplement: Source data 1. [file elife-64846-data1.zip › Raw_images/Figure_2/Panel_F_Tubulin .jpg]

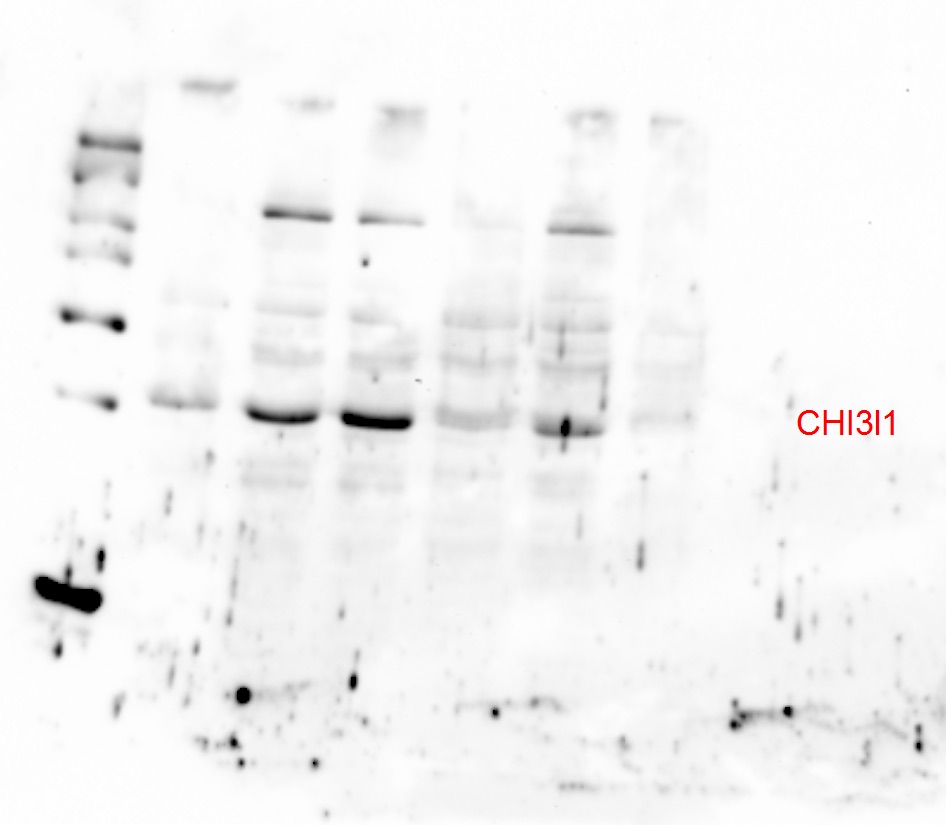

Supplement: Source data 1. [file elife-64846-data1.zip › Raw_images/Figure_2/Panel_F_CHI3L1.jpg]

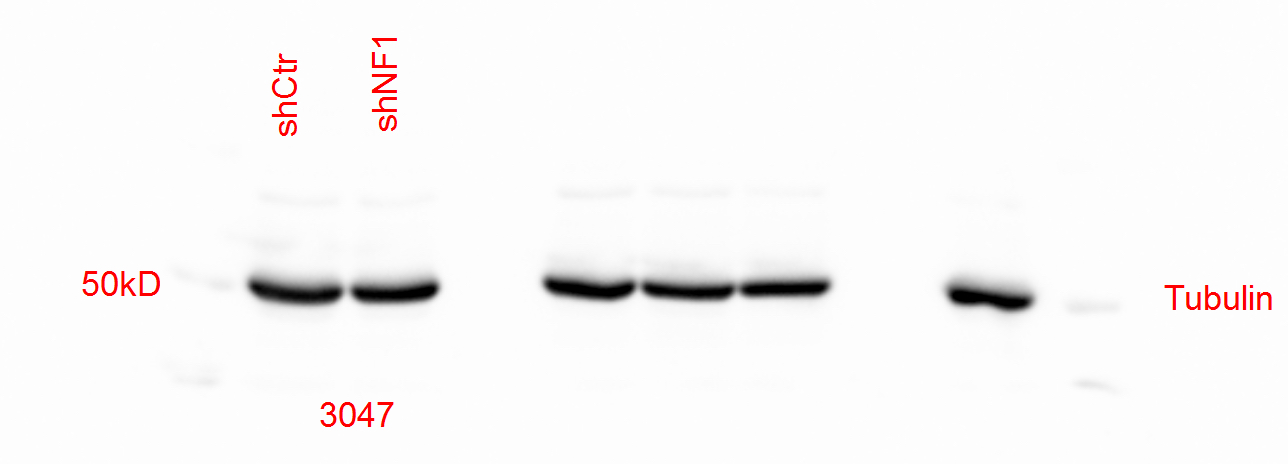

Supplement: Source data 1. [file elife-64846-data1.zip › Raw_images/Figure 2-figure supplement 2/Panel_E_right_shNF1_4_Tubulin.jpg]

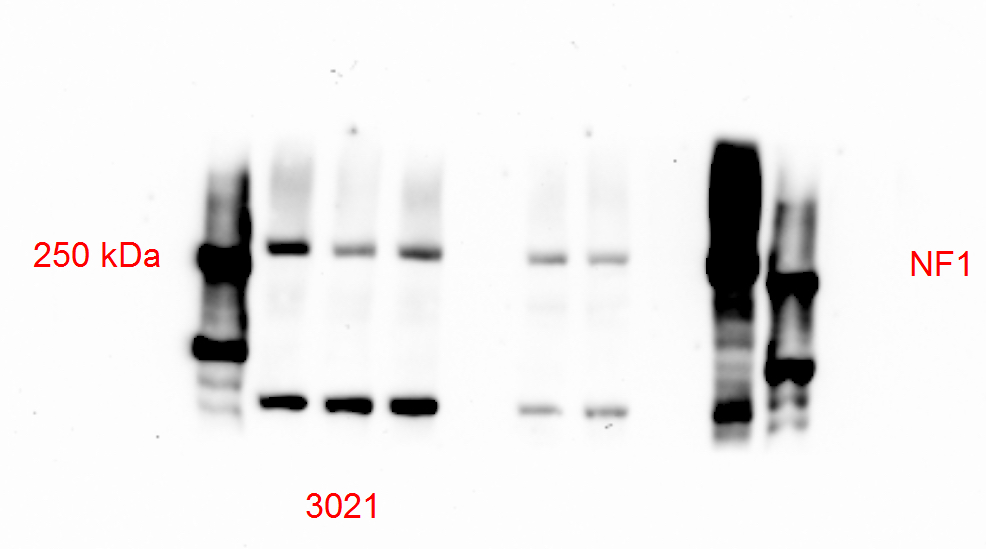

Supplement: Source data 1. [file elife-64846-data1.zip › Raw_images/Figure 2-figure supplement 2/Panel_E_left_shNF1_1_NF1.jpg]

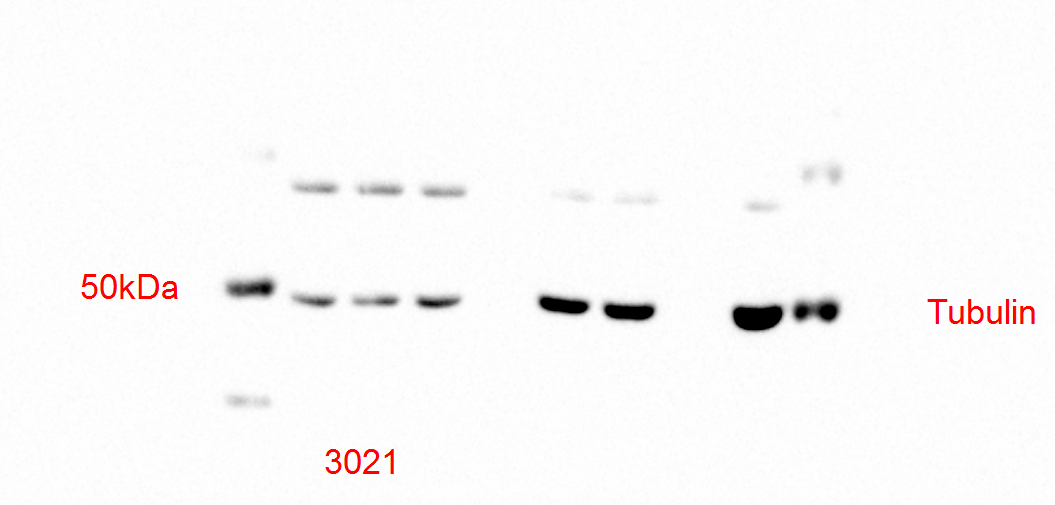

Supplement: Source data 1. [file elife-64846-data1.zip › Raw_images/Figure 2-figure supplement 2/Panel_E_left_shNF1_1Tubulin.jpg]

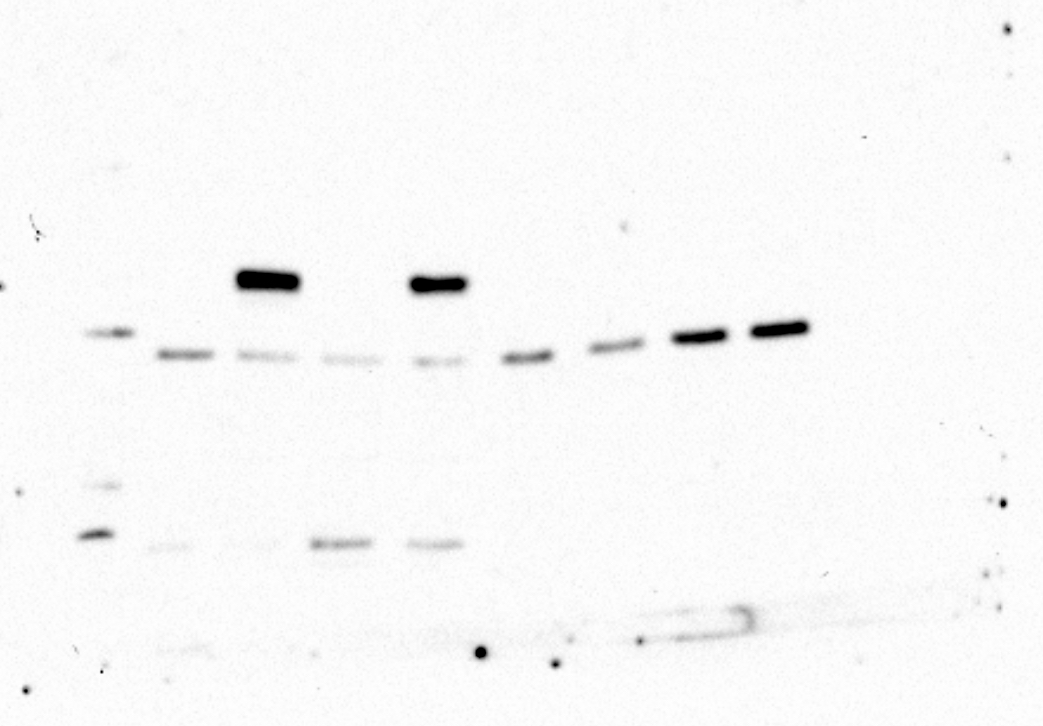

Supplement: Source data 1. [file elife-64846-data1.zip › Raw_images/Figure 2-figure supplement 2/Panel_J_FLAG-NF1-GRD.jpg]

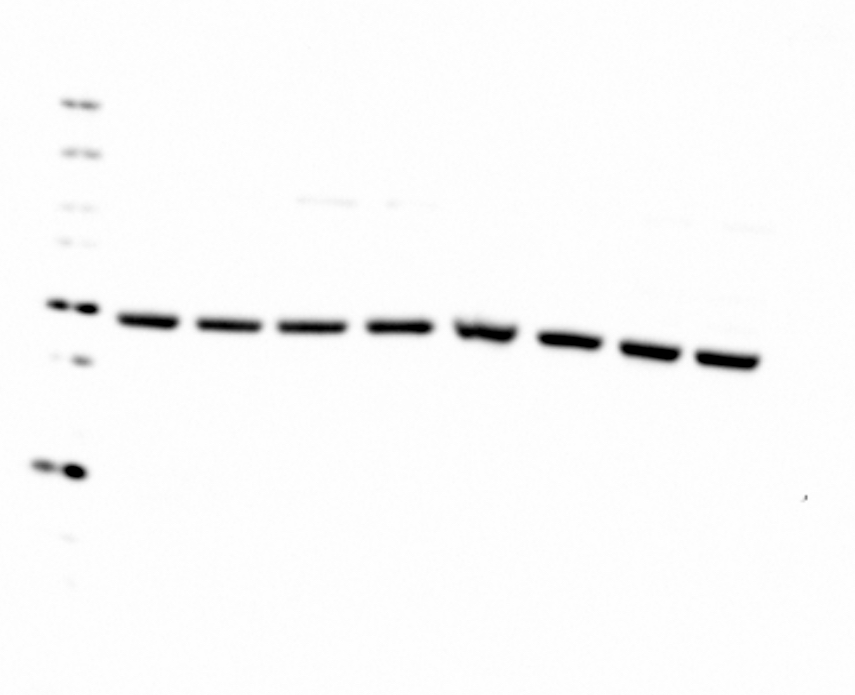

Supplement: Source data 1. [file elife-64846-data1.zip › Raw_images/Figure 2-figure supplement 2/Panel_J_Tubulin.jpg]

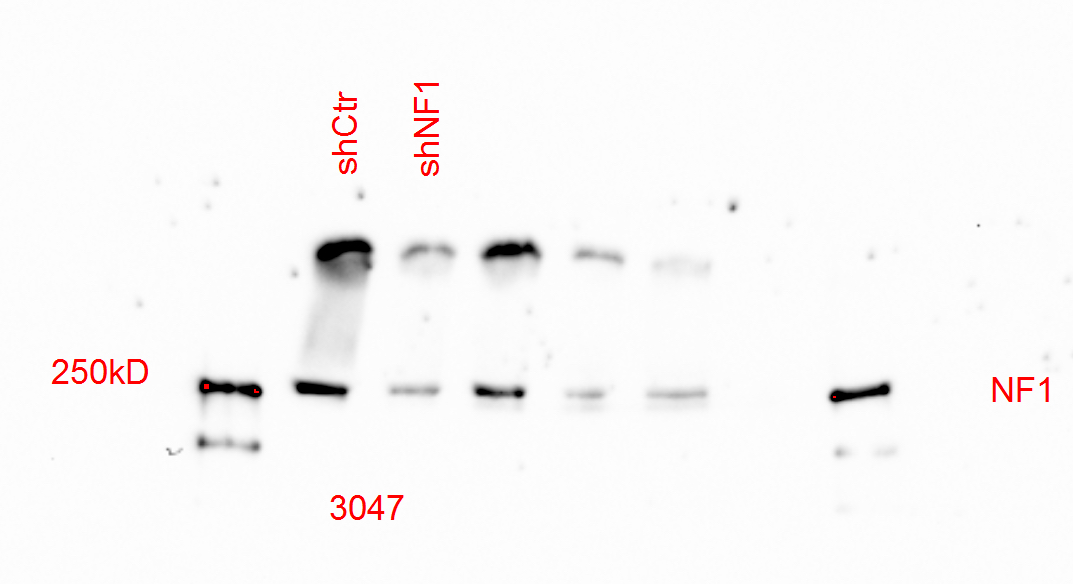

Supplement: Source data 1. [file elife-64846-data1.zip › Raw_images/Figure 2-figure supplement 2/Panel_E_right_shNF1_5_NF1.jpg]

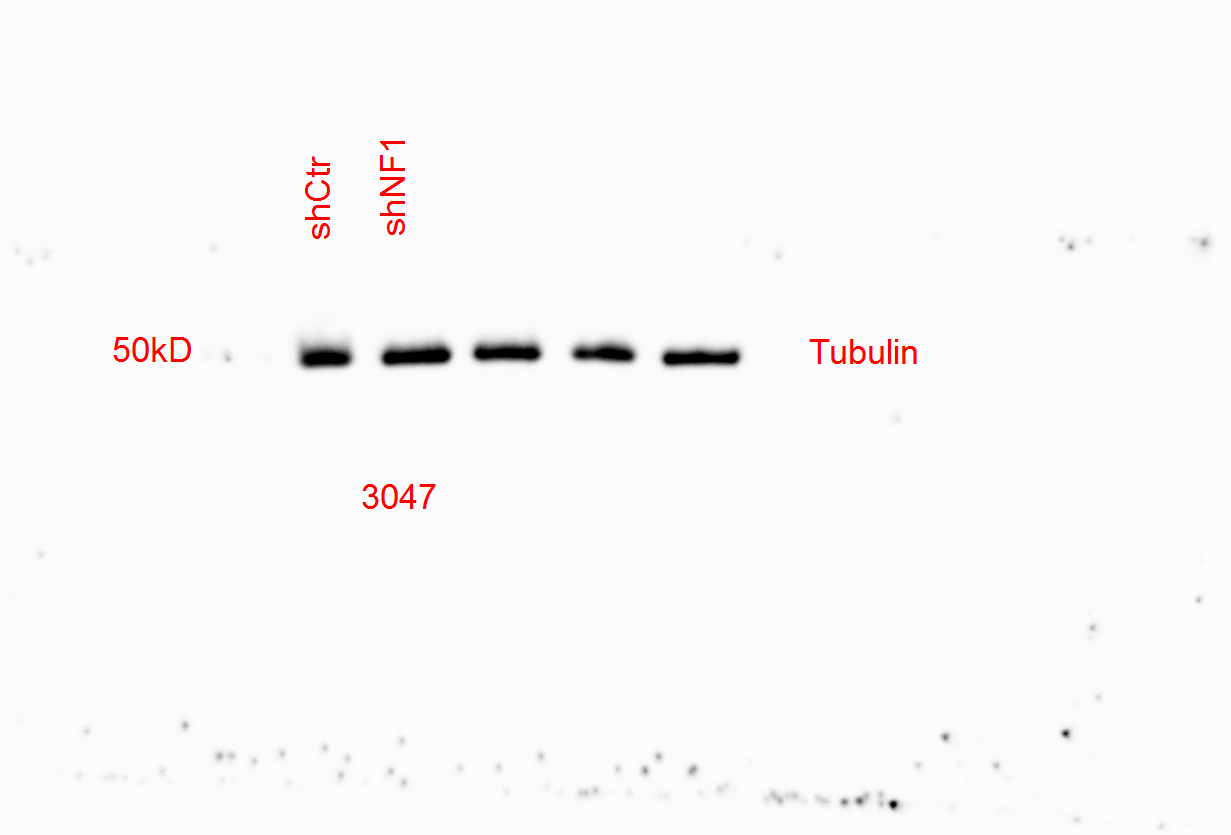

Supplement: Source data 1. [file elife-64846-data1.zip › Raw_images/Figure 2-figure supplement 2/Panel_E_right_shNF1_5_Tubulin.jpg]

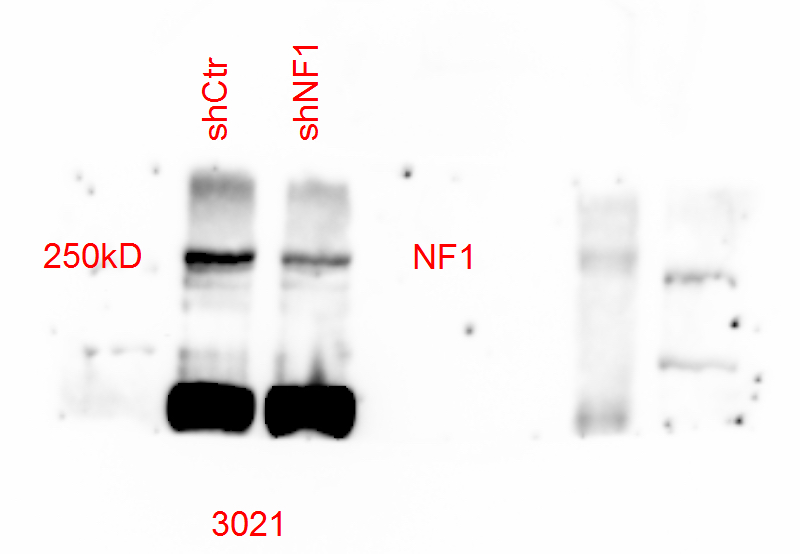

Supplement: Source data 1. [file elife-64846-data1.zip › Raw_images/Figure 2-figure supplement 2/Panel_E_left_shNF1_5_NF1.jpg]

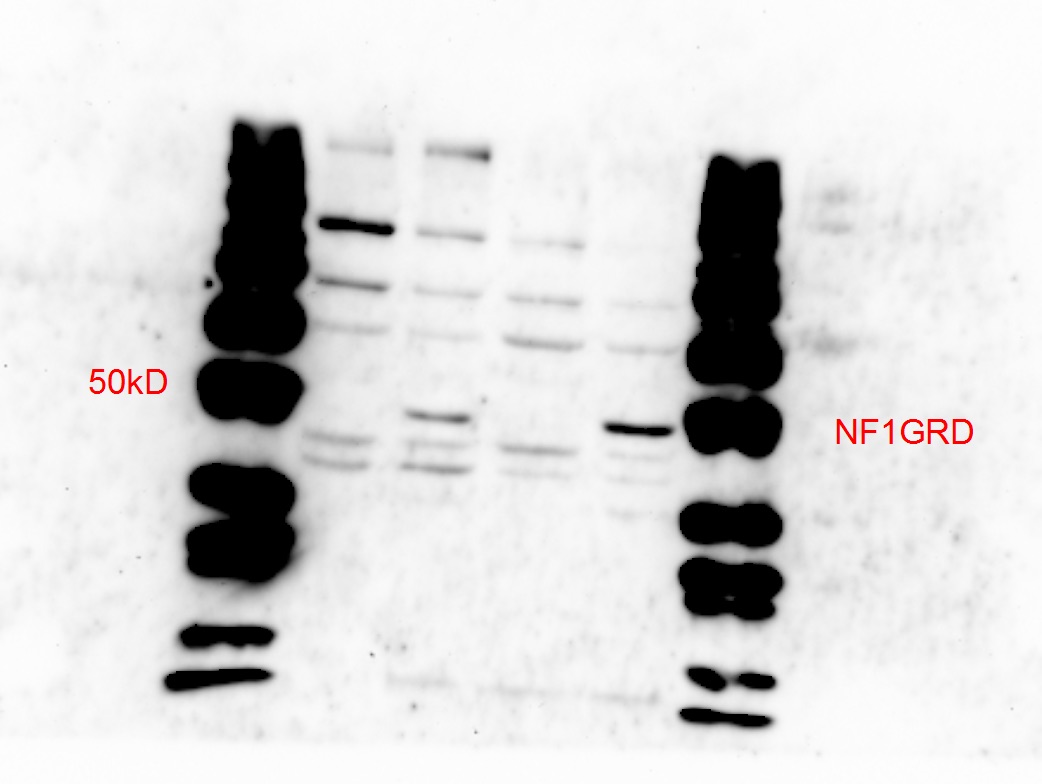

Supplement: Source data 1. [file elife-64846-data1.zip › Raw_images/Figure 2-figure supplement 2/Panel_A_Flag.jpg]

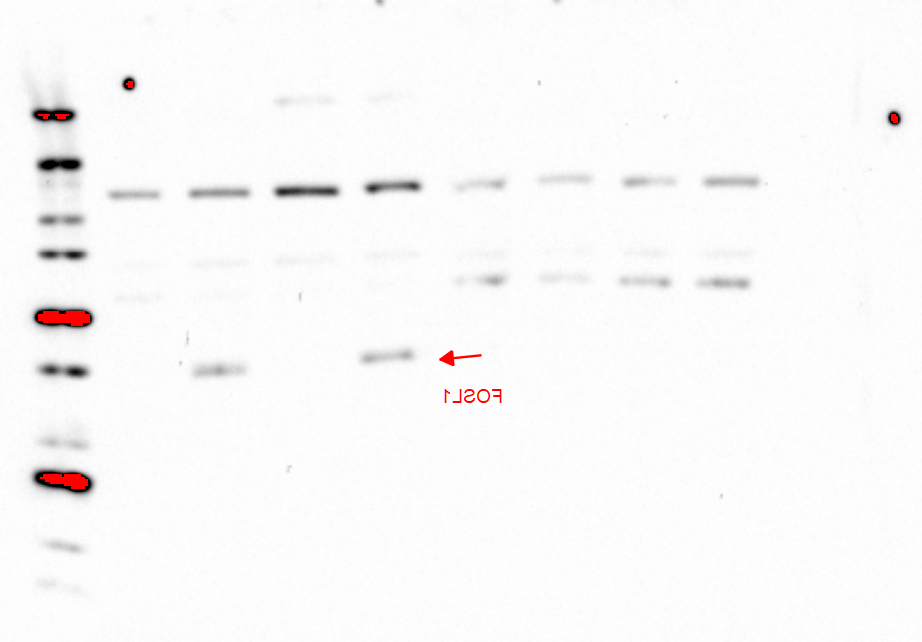

Supplement: Source data 1. [file elife-64846-data1.zip › Raw_images/Figure 2-figure supplement 2/Panel_J_FLAG-FOSL1.jpg]

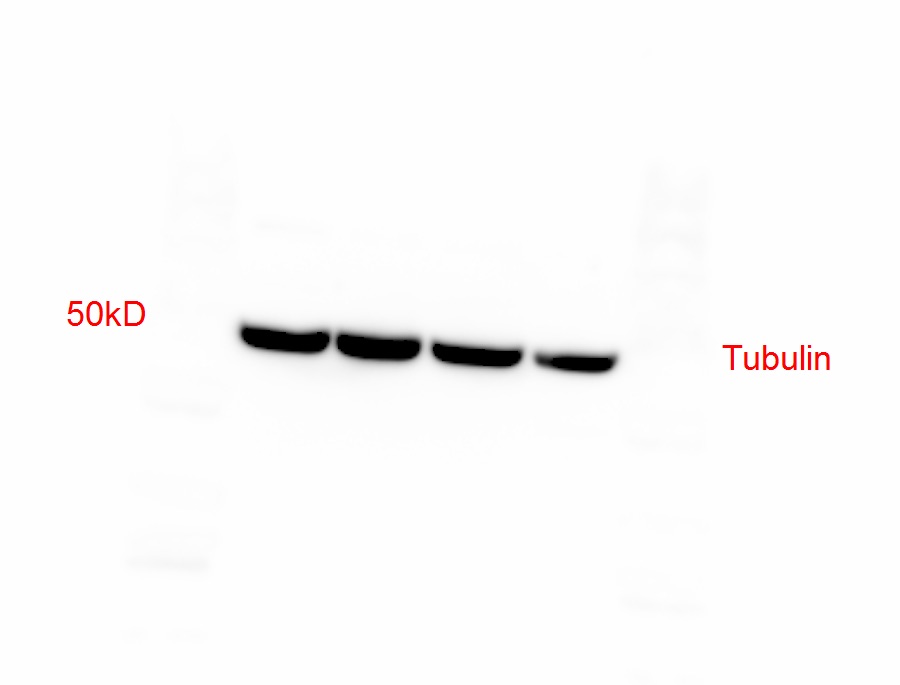

Supplement: Source data 1. [file elife-64846-data1.zip › Raw_images/Figure 2-figure supplement 2/Panel_A_Tubulin.jpg]

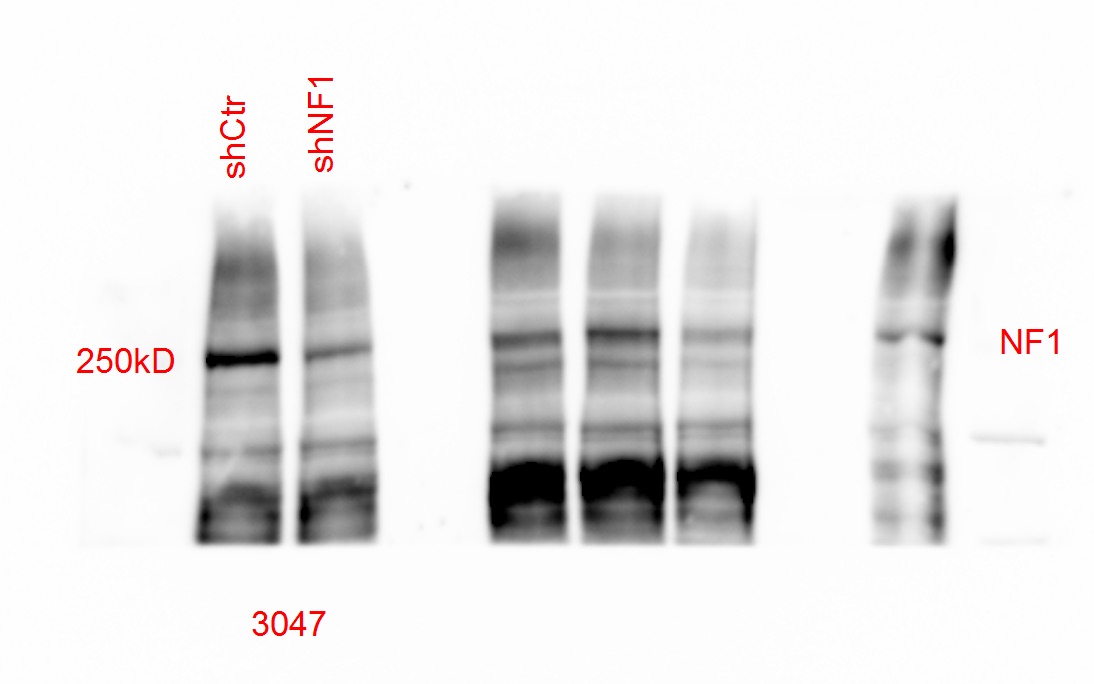

Supplement: Source data 1. [file elife-64846-data1.zip › Raw_images/Figure 2-figure supplement 2/Panel_E_right_shNF1_4_NF1.jpg]

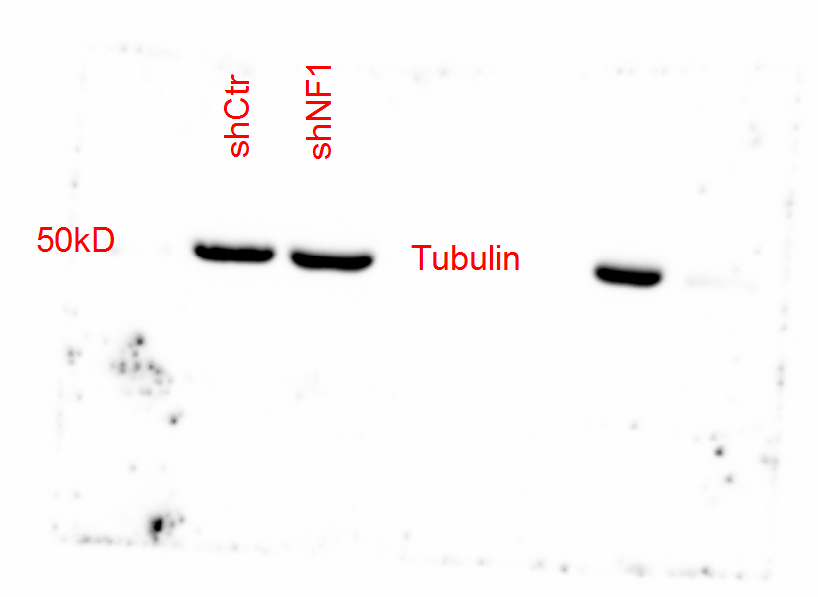

Supplement: Source data 1. [file elife-64846-data1.zip › Raw_images/Figure 2-figure supplement 2/Panel_E_left_shNF1_5Tubulin.jpg]

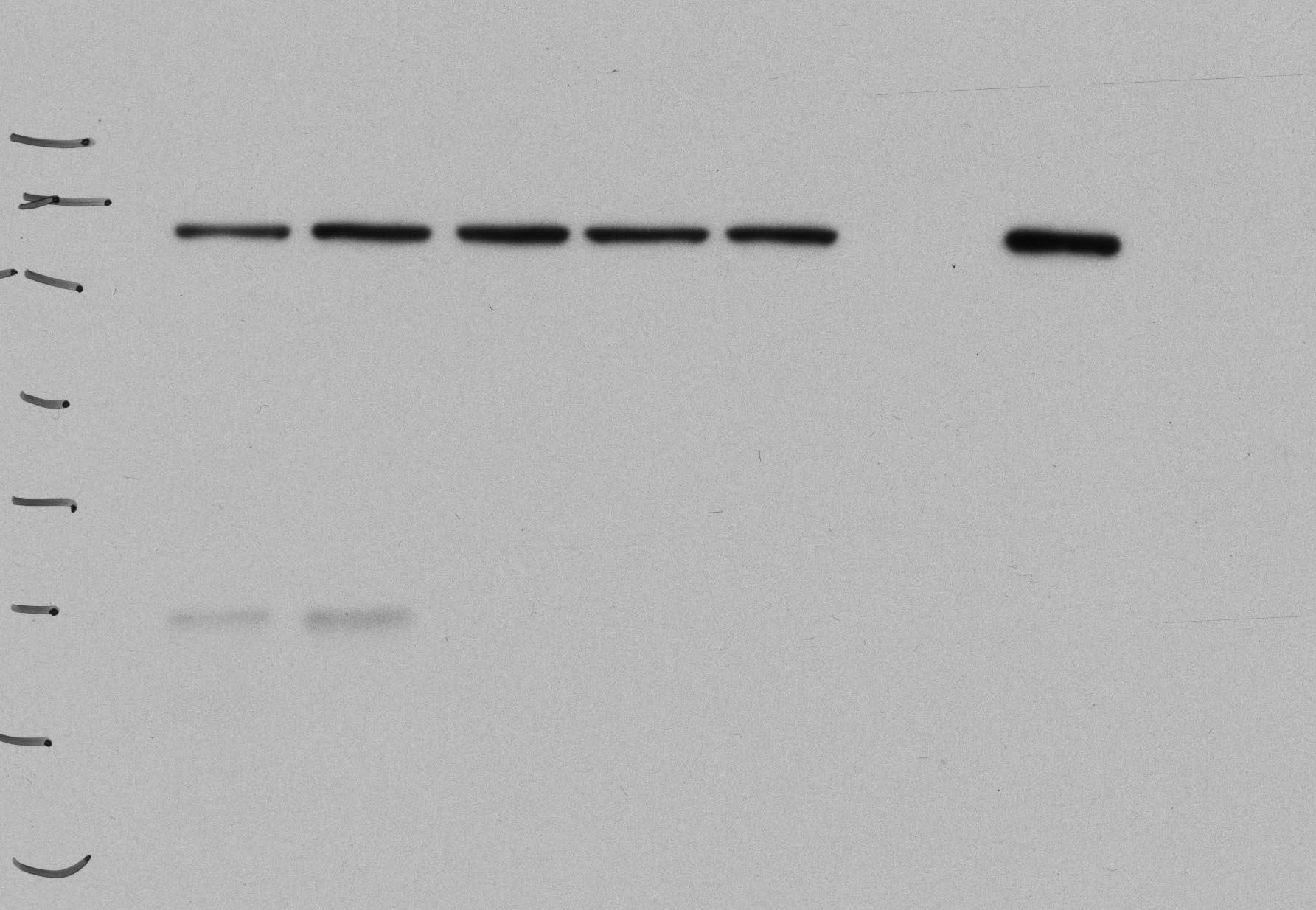

Supplement: Source data 1. [file elife-64846-data1.zip › Raw_images/Figure_3/Panel_C_vinculin.jpg]

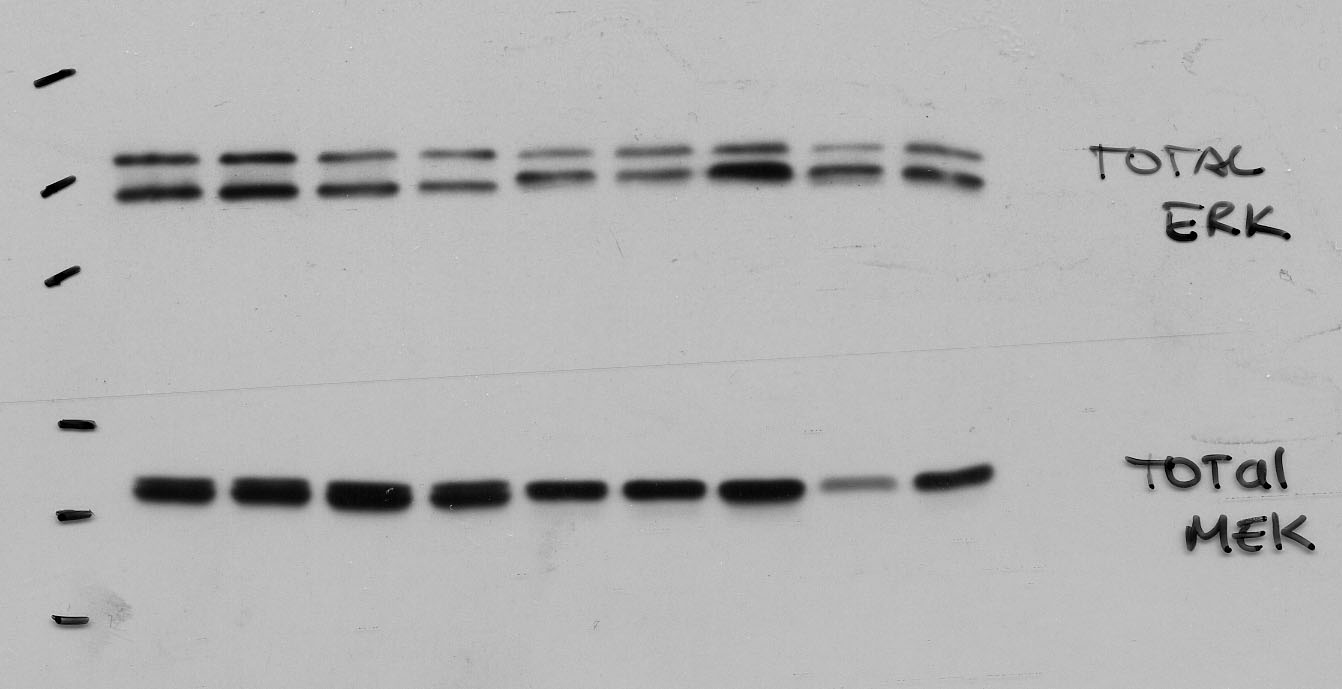

Supplement: Source data 1. [file elife-64846-data1.zip › Raw_images/Figure_3/Panel_A_ERK_MEK.jpg]

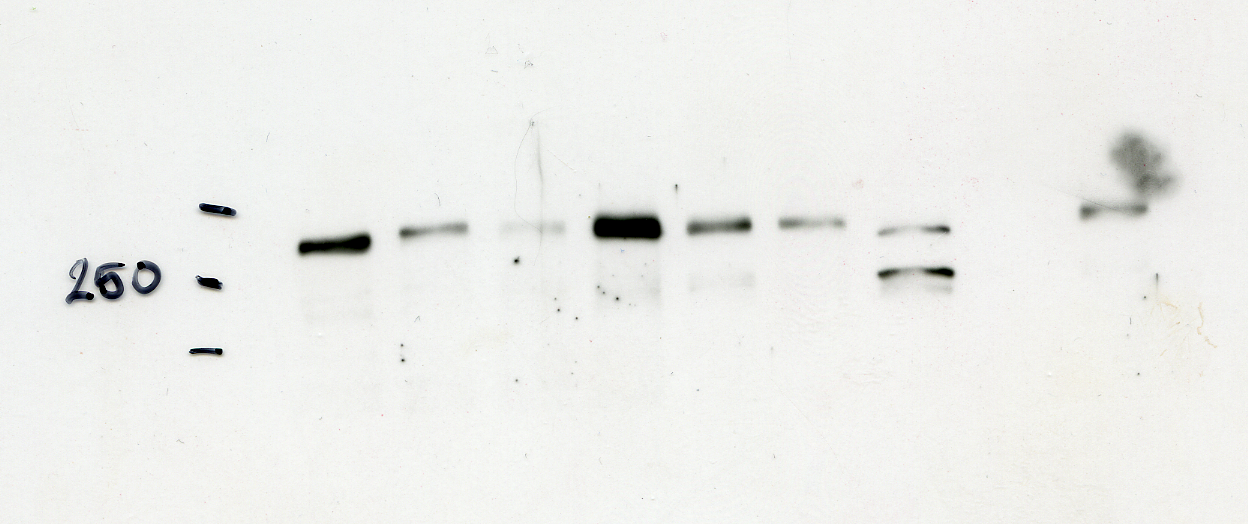

Supplement: Source data 1. [file elife-64846-data1.zip › Raw_images/Figure_3/Panel_A_NF1.tif]

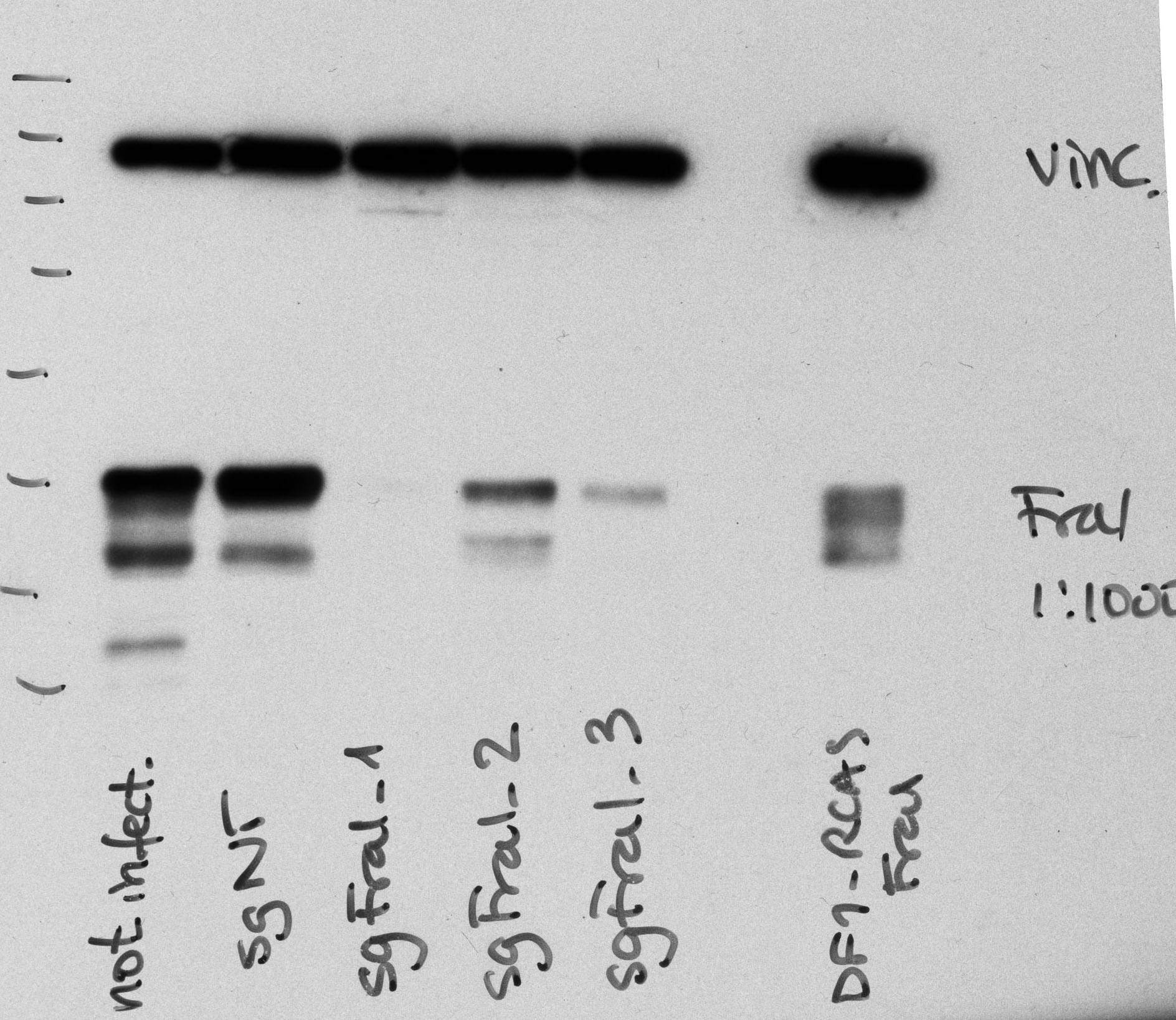

Supplement: Source data 1. [file elife-64846-data1.zip › Raw_images/Figure_3/Panel_C_FRA1.jpg]

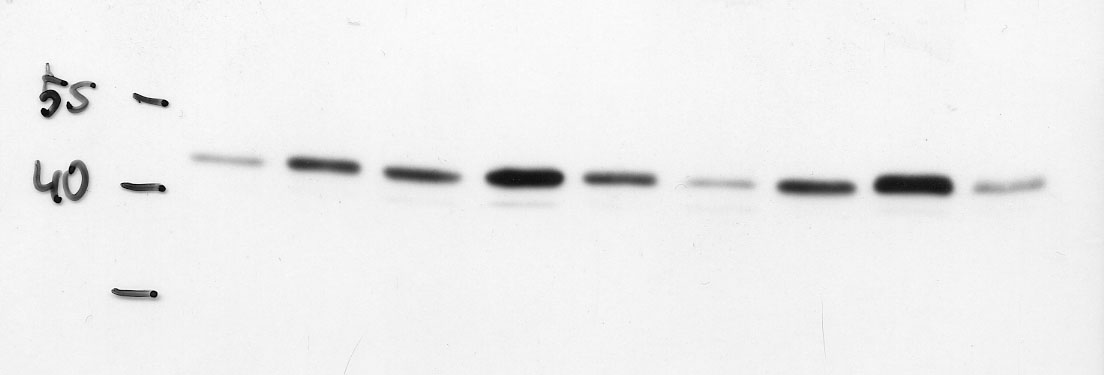

Supplement: Source data 1. [file elife-64846-data1.zip › Raw_images/Figure_3/Panel_A_pMEK.jpg]

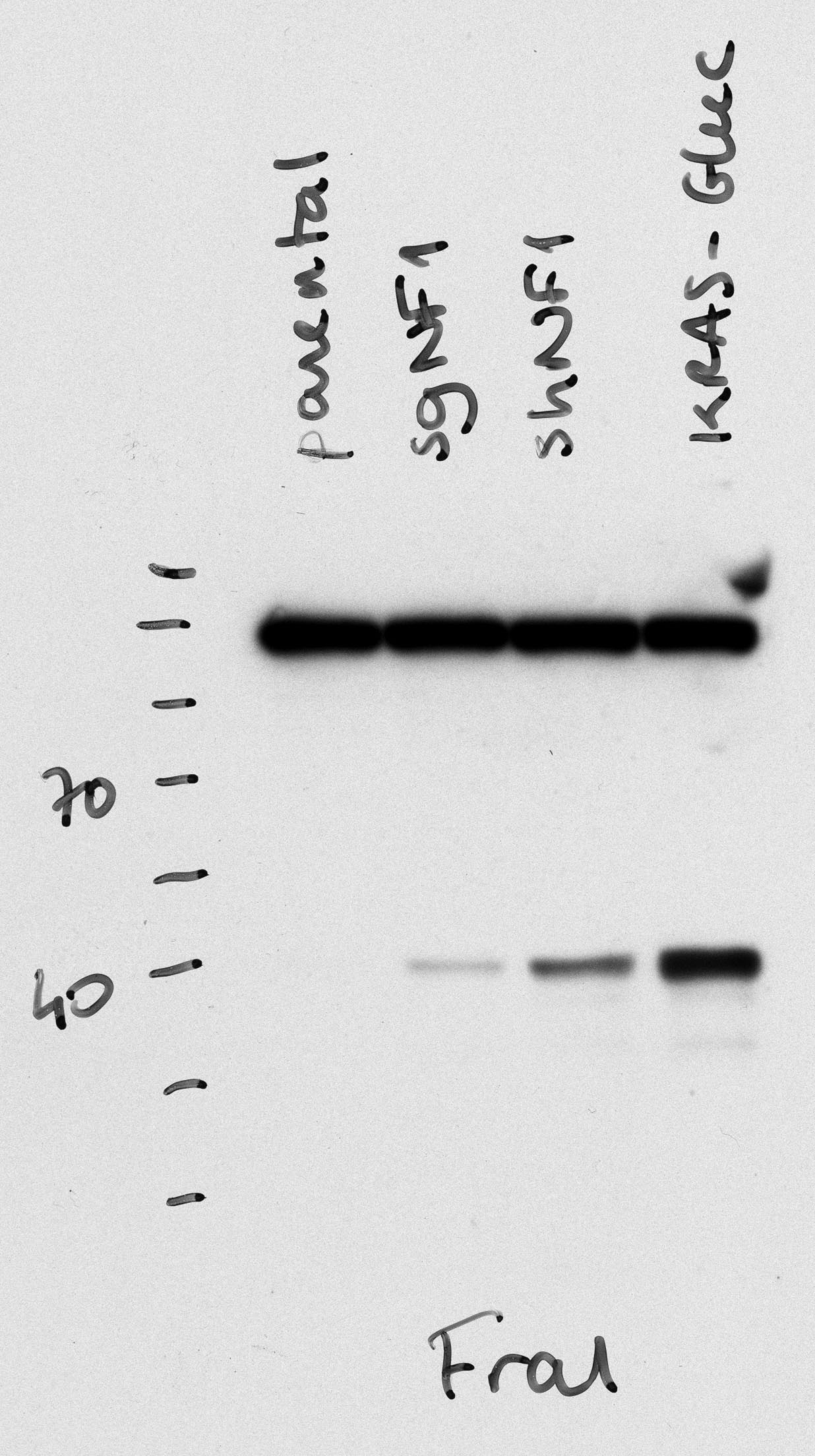

Supplement: Source data 1. [file elife-64846-data1.zip › Raw_images/Figure_3/Panel_A_FRA1.jpg]

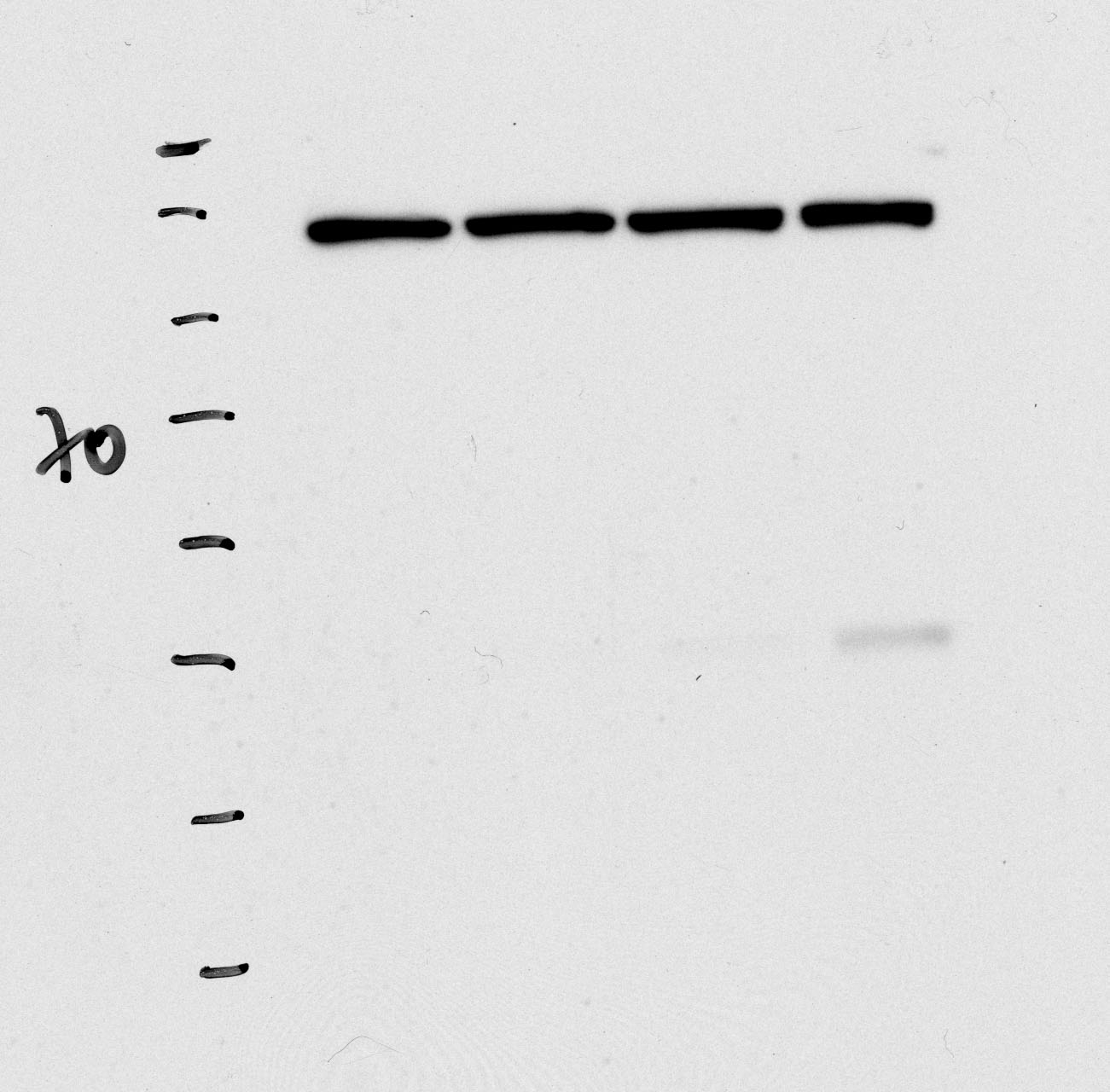

Supplement: Source data 1. [file elife-64846-data1.zip › Raw_images/Figure_3/Panel_A_vinculin.jpg]

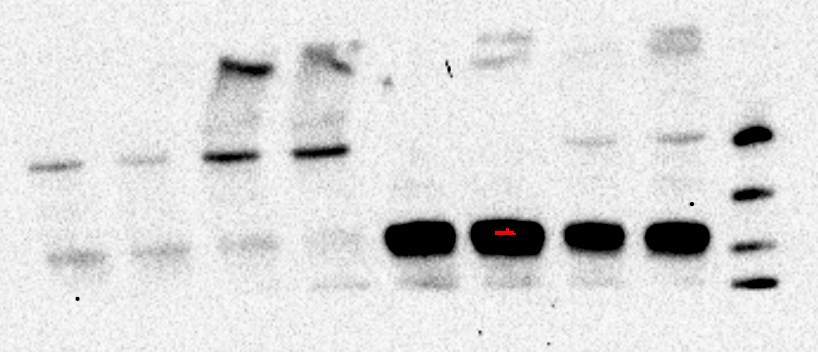

Supplement: Source data 1. [file elife-64846-data1.zip › Raw_images/Figure 2-figure supplement 3/Panel_A_NF1.jpg]

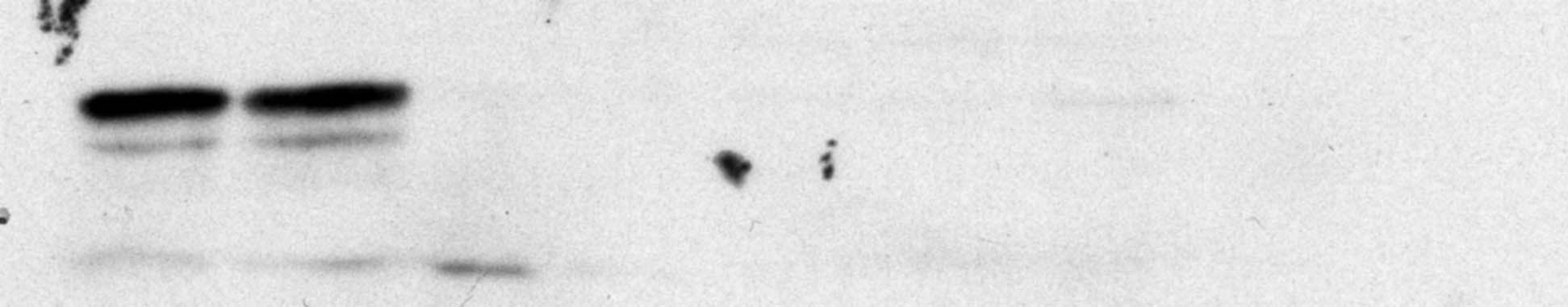

Supplement: Source data 1. [file elife-64846-data1.zip › Raw_images/Figure 2-figure supplement 3/Panel_E_FRA1.jpg]

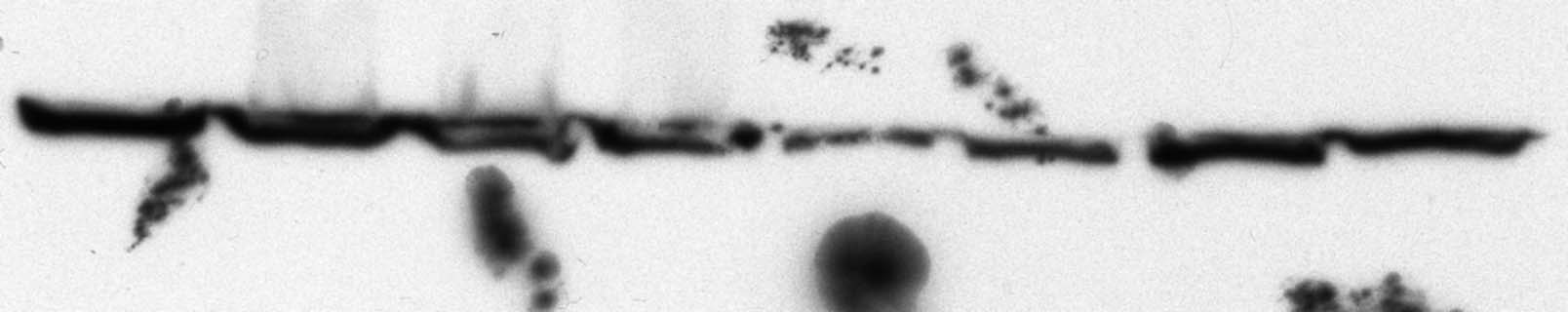

Supplement: Source data 1. [file elife-64846-data1.zip › Raw_images/Figure 2-figure supplement 3/Panel_E_vinculin.jpg]

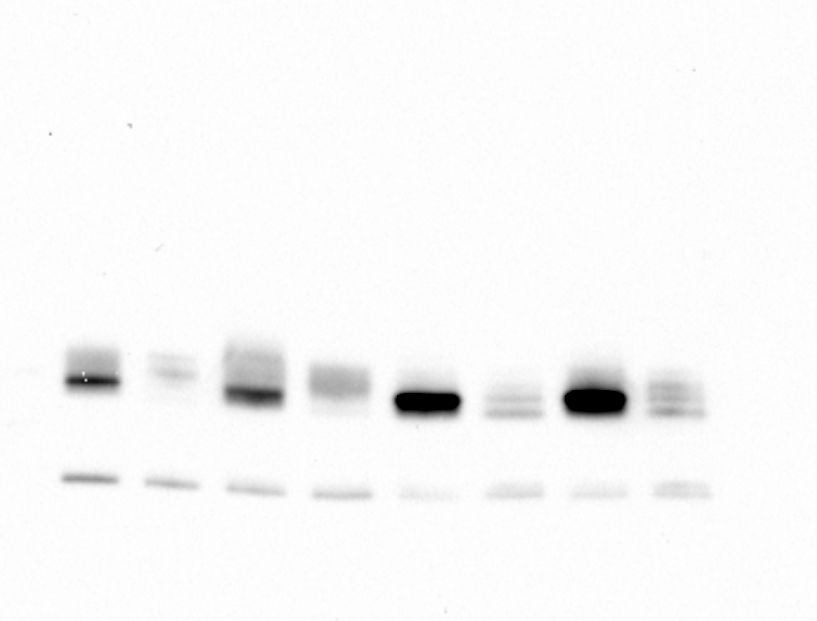

Supplement: Source data 1. [file elife-64846-data1.zip › Raw_images/Figure 2-figure supplement 3/Panel_A_pERK12.jpg]

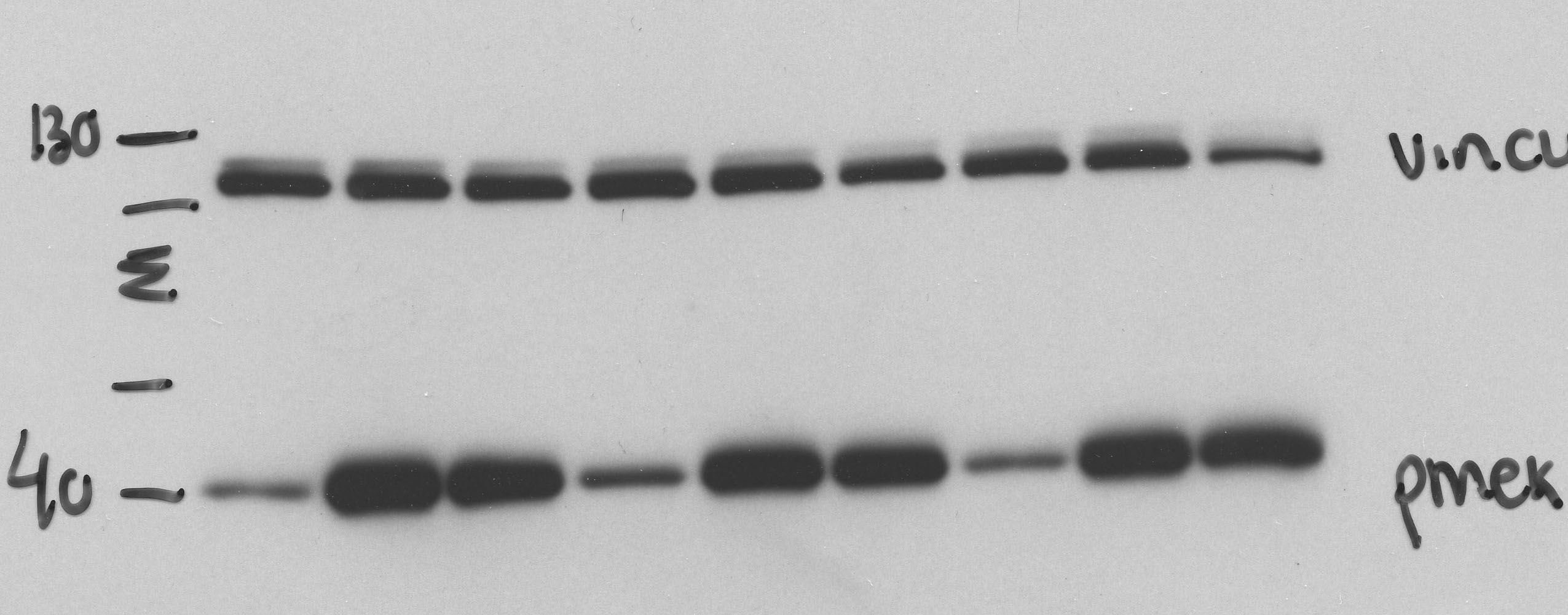

Supplement: Source data 1. [file elife-64846-data1.zip › Raw_images/Figure 2-figure supplement 3/Panel_C_pMEK_vinculin.jpg]

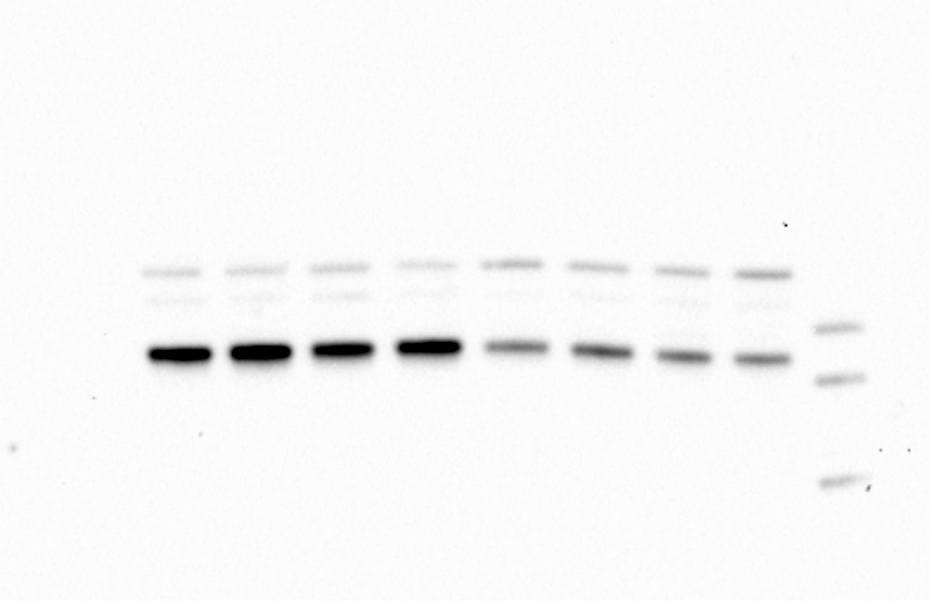

Supplement: Source data 1. [file elife-64846-data1.zip › Raw_images/Figure 2-figure supplement 3/Panel_A_MEK12.jpg]

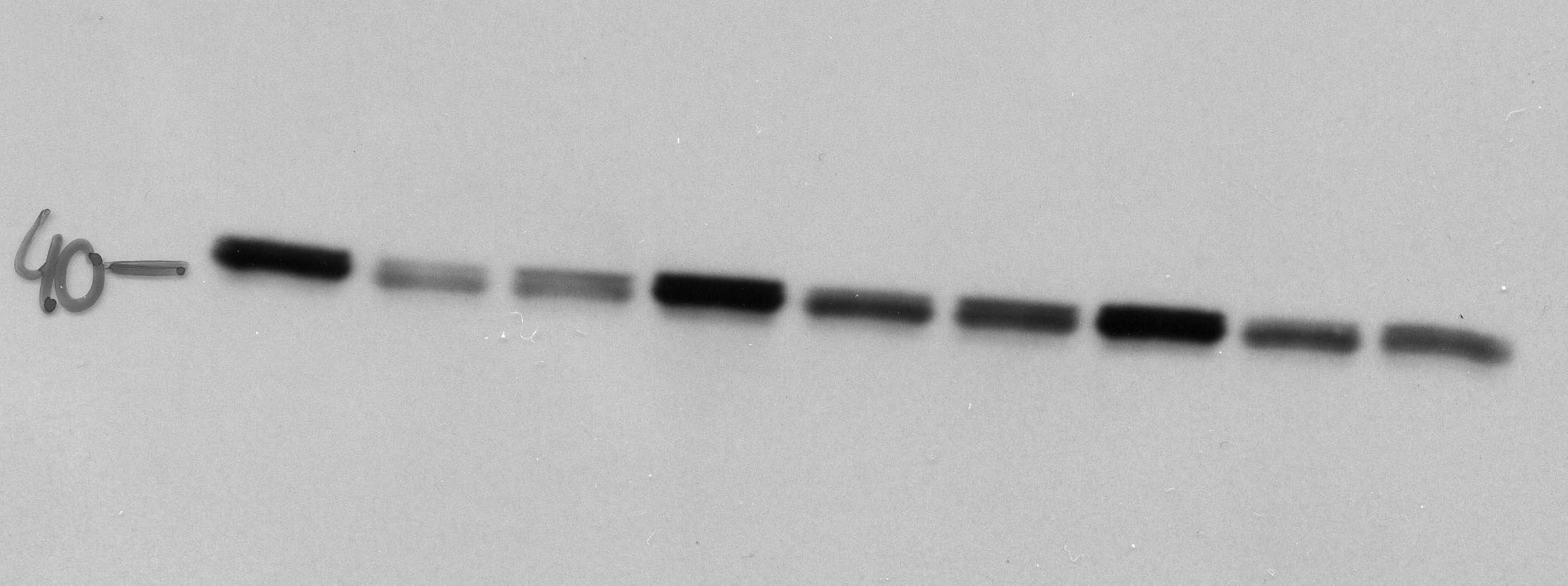

Supplement: Source data 1. [file elife-64846-data1.zip › Raw_images/Figure 2-figure supplement 3/Panel_C_TotMEK.jpg]

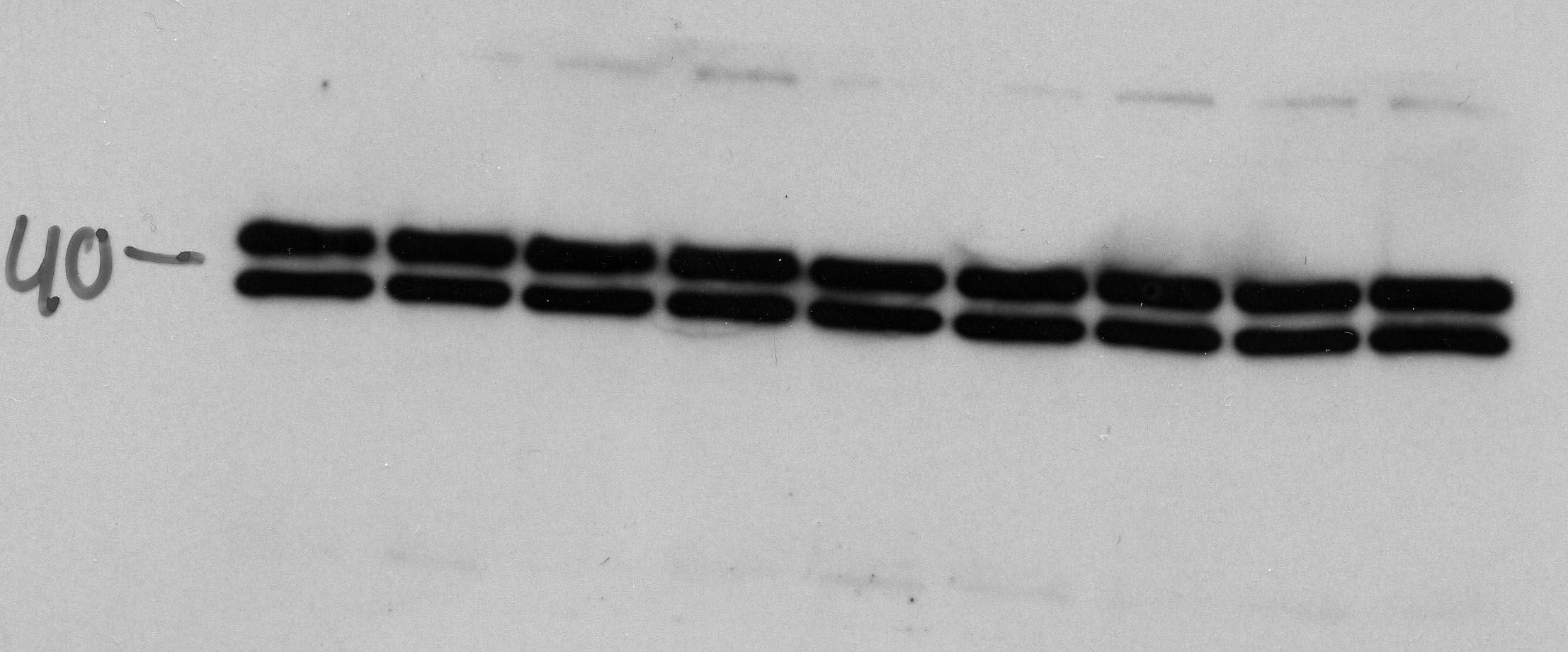

Supplement: Source data 1. [file elife-64846-data1.zip › Raw_images/Figure 2-figure supplement 3/Panel_C_TotERK.jpg]

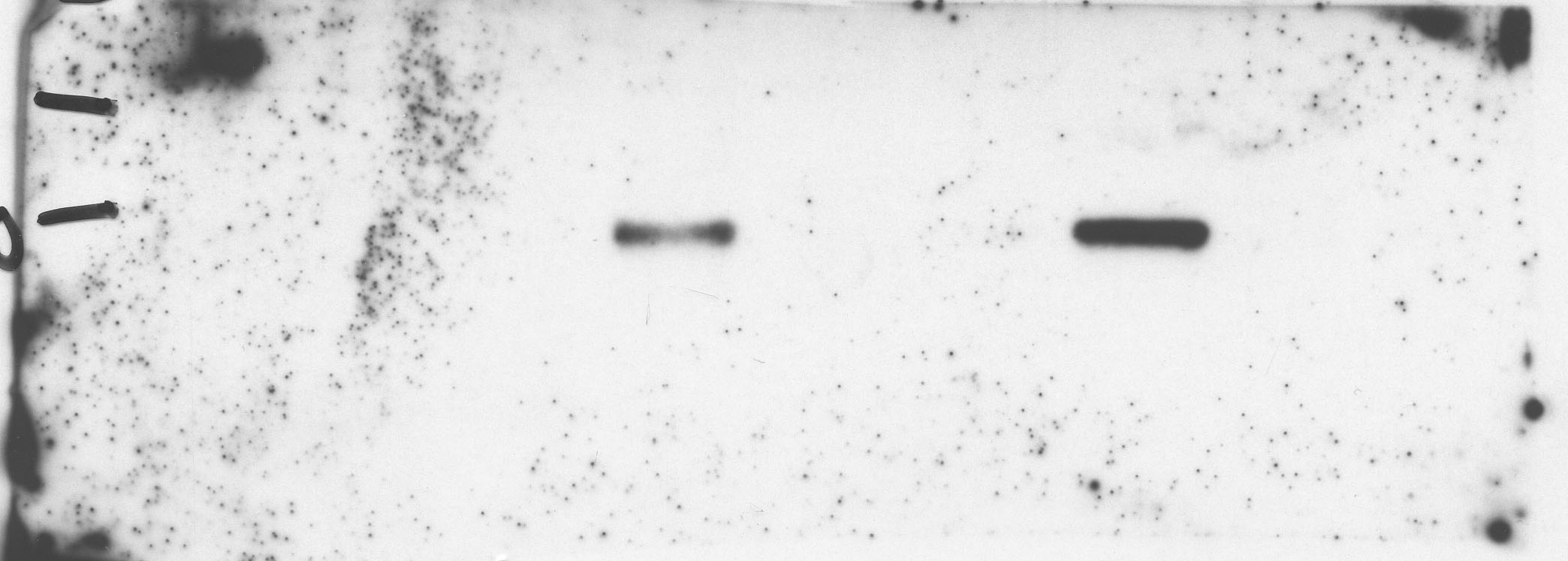

Supplement: Source data 1. [file elife-64846-data1.zip › Raw_images/Figure 2-figure supplement 3/Panel_C_FRA1]

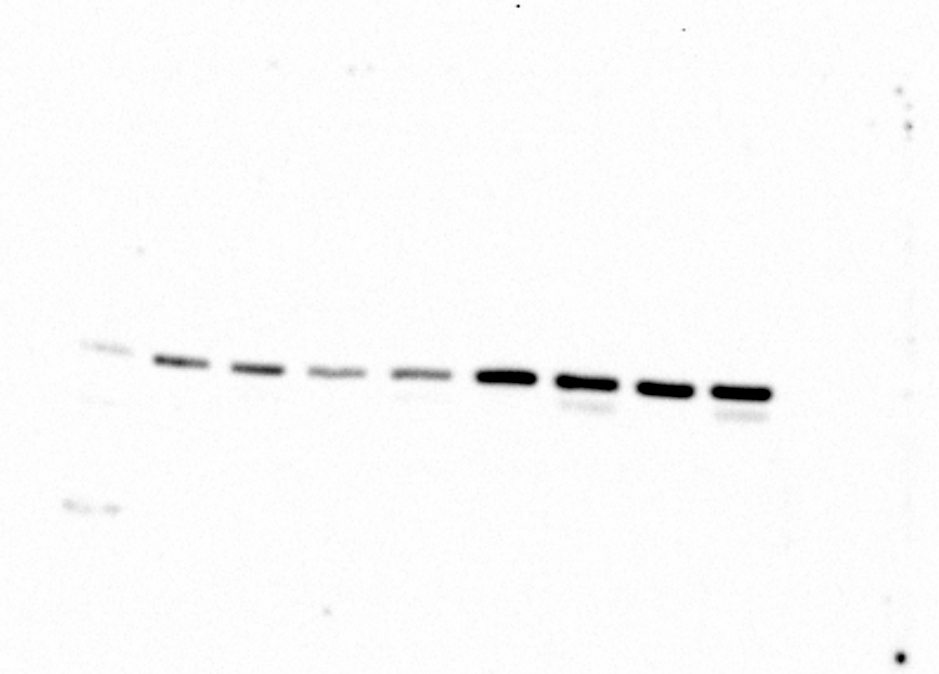

Supplement: Source data 1. [file elife-64846-data1.zip › Raw_images/Figure 2-figure supplement 3/Panel_A_tubulin.jpg]

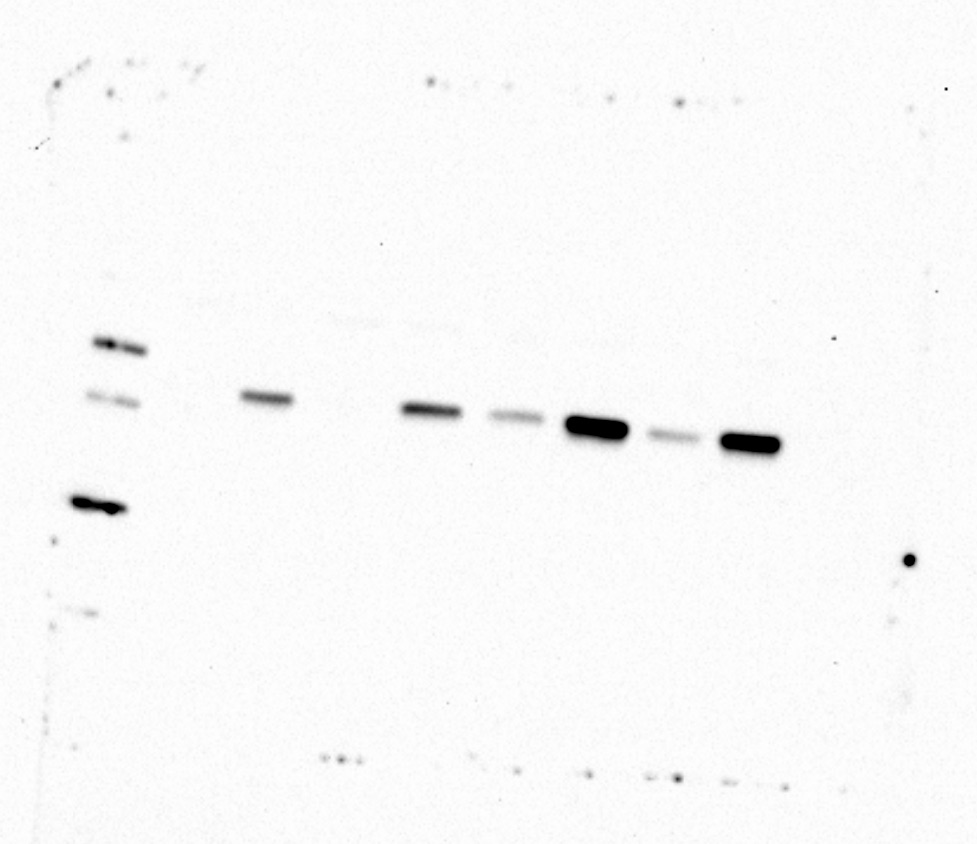

Supplement: Source data 1. [file elife-64846-data1.zip › Raw_images/Figure 2-figure supplement 3/Panel_A_pMEK12.jpg]

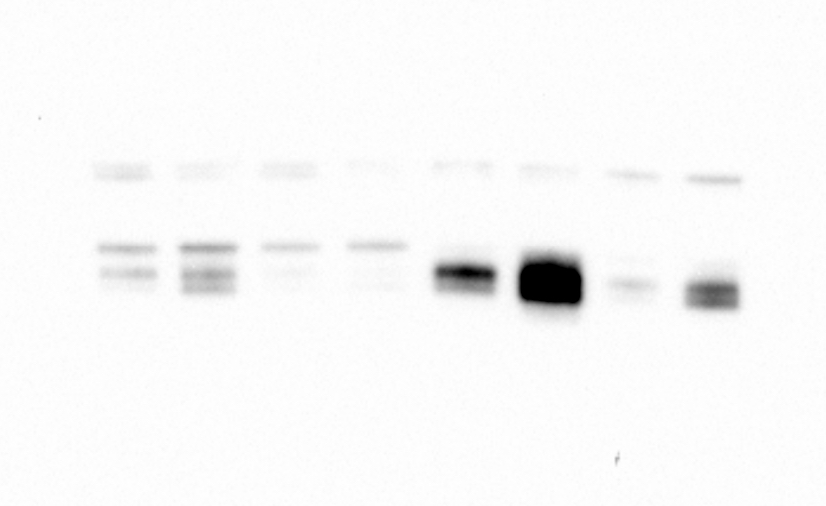

Supplement: Source data 1. [file elife-64846-data1.zip › Raw_images/Figure 2-figure supplement 3/Panel_A_FRA1.jpg]

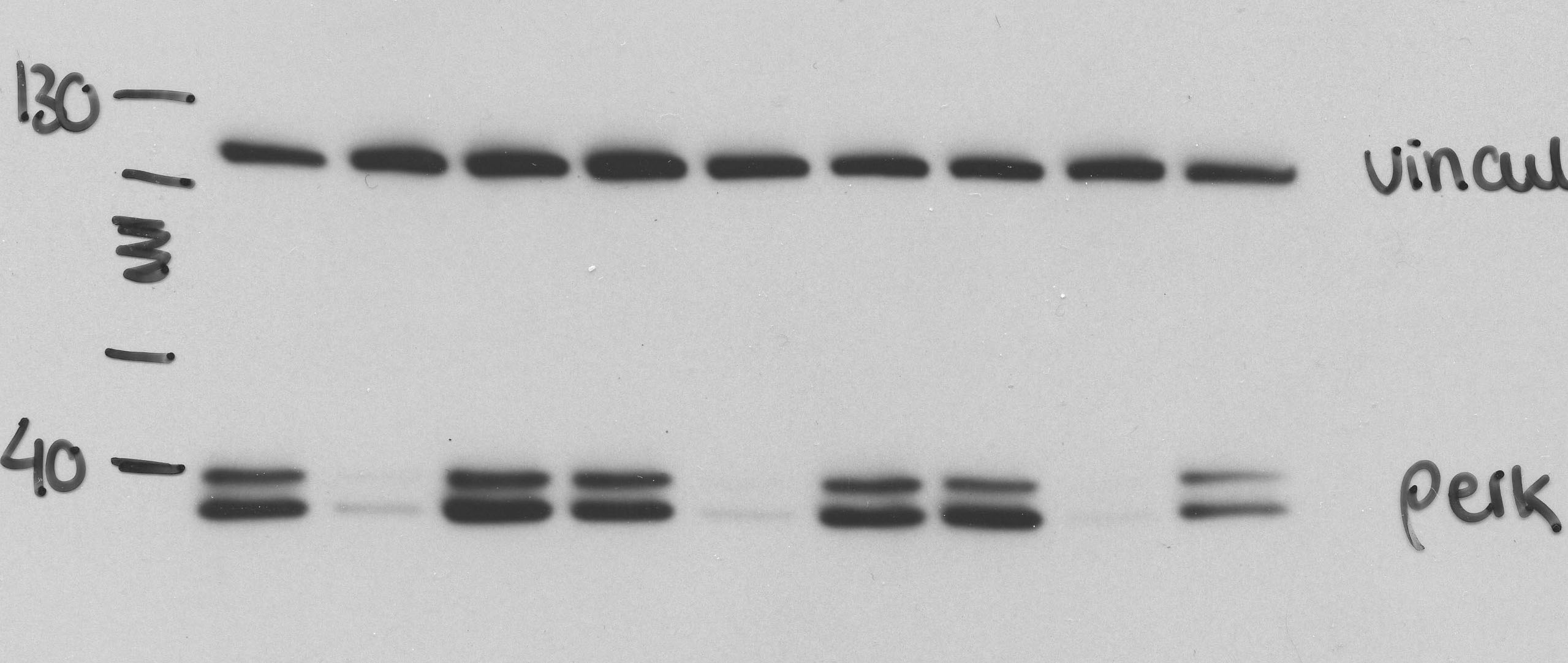

Supplement: Source data 1. [file elife-64846-data1.zip › Raw_images/Figure 2-figure supplement 3/Panel_C_pERK_vinculin.jpg]

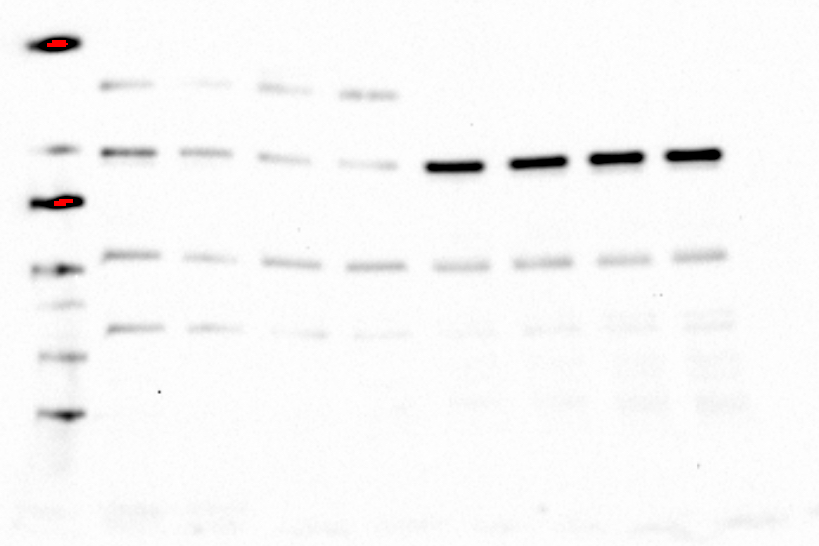

Supplement: Source data 1. [file elife-64846-data1.zip › Raw_images/Figure 2-figure supplement 3/Panel_A_ERK12.jpg]
